# Effectiveness of Native and Enhanced Extracellular Vesicle Preparations Assessed in Animal Models of Multiple Sclerosis: A Meta-Analysis

**DOI:** 10.3390/biom16101454

**Published:** 2026-10-05

**Authors:** Melania Magercu, Violeta Ristoiu, Bogdan Amuzescu, Adriana Octaviana Dulămea

**Affiliations:** 1 Department of Anatomy, Animal Physiology and Biophysics, Faculty of Biology, University of Bucharest, Splaiul Independentei 91-95, 050095 Bucharest, Romania; melania-maria.magercu@bio.unibuc.ro (M.M.); violeta.ristoiu@bio.unibuc.ro (V.R.); 2 “Carol Davila” University of Medicine and Pharmacy, 37 Dionisie Lupu Street, 020021 Bucharest, Romania; octaviana.dulamea@umfcd.ro or adrianadulamea@gmail.com; 3 Department of Neurology, Fundeni Clinical Institute, 258 Fundeni Street, 022328 Bucharest, Romania

**Keywords:** animal model, extracellular vesicles, multiple sclerosis, demyelination, inflammation, clinical score, pathological score

## Abstract

Extracellular vesicle (EV) preparations have emerged as potentially useful therapeutic tools for various inflammatory or neurodegenerative CNS disorders. Compared to mesenchymal stem cell preparations, they feature enhanced blood–brain barrier permeability and access to injured brain tissue, and better quality assessment and dosing options. A particular case where such therapies may prove beneficial is multiple sclerosis (MS), an autoimmune disease likely triggered by cross-reactivity with some viral antigens, producing an extended demyelinating encephalomyelitis with multiple patchy lesions. Several animal models of MS have been developed, including experimental autoimmune encephalomyelitis (EAE) via injection of myelin oligodendrocyte glycoprotein (MOG) 35–55, inoculation with Theiler’s murine encephalomyelitis virus (TMEV), and the Cuprizone model. Via literature search we retrieved 24 experimental studies on rodent (rat or mouse) MS models with therapeutic administration of native or enhanced EV preparations suitable for this meta-analysis. We found statistically significant mean differences between experimental and control groups for clinical scores (normalized to a 0–5 scale) (−1.21, 95% confidence interval (CI) [−1.49,−0.93] (native EVs); −1.67, CI [−2.28,−1.05] (enhanced EVs)), for pathological scores of inflammation and demyelination, and for several molecular markers of inflammation and myelin repair. These results point to the need for clinical translation of the encouraging preliminary results obtained using such MS animal models.

## 1. Introduction

Multiple sclerosis (MS) is a demyelinating disease of the brain and the spinal cord with multiple patchy lesions resulting in glial scars, first described histologically by Jean-Martin Charcot, who named the disease “sclérose en plaques” [1]. It is an autoimmune disease triggered by unknown stimuli, on a background involving many genetic and environmental factors [2]. However, cross-reactivity with self-antigens following viral or bacterial infections seems to be the most plausible mechanism [3]. Epstein–Barr virus (EBV) appears to be a likely trigger of autoimmunity in MS, since Epstein–Barr nuclear antigen 1—EBNA1—is cross-reactive with GlialCAM—a cell adhesion molecule [4]. These viral antigens act in combination with genetic factors such as peculiar HLA variants (increased risk with DR15, protective effects with DRB1*B11 and C554) [5], contributing to a total of 551 candidate risk genes that account for the polygenic susceptibility component of the disease [6]. A recent study proved that anti-CD20 antibody therapy in MS patients resulted in a reduction in both EBV-specific CD4+ T and B lymphocyte clones [7].

The CNS-reactive T cells reaching the brain in early phases reactivate in contact with the self-antigens and trigger neuroinflammation with massive monocyte/macrophage infiltration and recruitment of microglia, the main effectors of demyelination, leading to axonal injury and loss [8]. This phenomenon is exacerbated by the encephalitogenic T cell–microglia cross-talk [9].

To investigate the etiopathogenesis of MS, several animal models of MS have been developed, including experimental autoimmune encephalomyelitis (EAE) induced by injection of myelin oligodendrocyte glycoprotein (MOG) 35–55 (a minor component of CNS myelin); intracerebral inoculation with Theiler’s murine encephalomyelitis virus (TMEV); a surrogate model involving treatment with the copper chelating agent biscyclohexanone oxaldihydrazone (Cuprizone or CPZ), which inhibits the mitochondrial cytochrome oxidase complex selectively in oligodendrocytes; and direct injection of lysophosphatidylcholine or ethidium bromide into white matter tracts [10].

Current MS treatment includes management of acute relapses, symptomatic treatment and disease-modifying therapies (DMTs) aimed primarily at controlling inflammatory disease activity. DMTs encompass immunomodulatory and immunosuppressive agents with different mechanisms of action, which can substantially reduce relapse frequency and delay disability progression. However, despite major advances in controlling neuroinflammation, their capacity to promote effective remyelination and repair established CNS damage remains limited [11]. Consequently, increasing attention has been directed toward therapeutic strategies combining immunomodulatory effects with neuroprotective and regenerative potential.

Among therapies used for several autoimmune diseases and various CNS pathologies are mesenchymal stem cells (MSCs). The term *stem cell* (in German, *Stammzelle*) was first formulated by the famous 19th century biologist Ernst Haeckel by analogy with *Stammbäume* (stem trees or genealogy/phylogenetic trees), meant to designate the primordial unicellular organism from which all multicellular species evolved. Later on, the term was transferred to the fertilized egg or zygote, the origin of all differentiated cells of an organism [12]. The name was used during late 19th century by Theodor Boveri and Valentin Häcker to describe the germline cell lineage carrying the germplasm, but also by Häcker to describe the unique cell giving rise to the mesoderm, and by researchers like Pappenheim, Maximow and Ernst Neumann for cells giving rise to the cellular components of blood, i.e., hematopoietic stem cells [13]. Subsequent studies by Till, McCulloch and Becker in the early 1960s established the existence of common hematopoietic stem cells [14,15].

As a minimal definition, stem cells are cells endowed with a capacity for self-renewal, proliferation and differentiation into one or several specialized cell types [16]. The cells of the early stages of development of an embryo can be classified as totipotent (zygote and morula), pluripotent (blastocyst inner cell mass, capable of producing derivatives of all three germ layers), multipotent (differentiating into multiple cell types derived from a single germ layer), oligopotent or unipotent (progenitor or committed stem cells) [17,18]. Inner cell mass can be extracted, dissociated and cultured in vitro, producing *embryonic stem cells* (ESCs). Since 2006, the technique developed by Yamanaka and Takahashi [19] has allowed in vitro reprogramming of adult differentiated cells into *induced pluripotent stem cells* (iPSCs) [20,21].

*Mesenchymal stem cells* (MSCs) represent an important therapeutic resource. They can have different origins: bone marrow (BM-MSCs, not to be confounded with bone marrow stromal cells—BMSCs), umbilical cord and umbilical cord blood (UC-MSCs and UCB-MSCs), adipose tissue (AdMSCs), menstrual blood (MBSCs), placenta, amniotic fluid (AFSCs), and other tissues like dental pulp [16]. Minimal definition criteria of MSCs include a “stromal” origin, potential to proliferate and form colonies in vitro (which may be tested with the fibroblast colony-forming unit assay), adherence to plastic dishes, a spindle shape [22] and the capacity to differentiate in appropriate conditions into multiple mesoderm derivatives such as osteoblasts, chondroblasts and adipocytes [23]. MSCs can be characterized according to a set of specific markers: they are positive for surface antigens including CD73, CD90, CD105, integrins (CD29, CD49e), and cell adhesion molecules (CD31, CD44, CD54), and negative for CD34, CD45, CD79a, CD117, CD133, and class II HLA like HLA-DR [24].

MSCs have been tested intensively in various animal models and clinical trials for diseases of the central nervous system (CNS) such as traumatic brain injury, spinal cord injury and ischemic stroke [25]. Beyond their capacity for transdifferentiation [26], MSCs exert anti-inflammatory, antiapoptotic and regenerative effects via complex mechanisms, including secretion of anti-inflammatory cytokines such as IL-4 and IL-10, activation of the NF-κB pathway leading to increased IL-6 production and improved viability of neural stem cells [27]. A review of early clinical trials of MSCs for ischemic stroke treatment described multiple types of preparations, mostly allogeneic or autologous bone marrow MSCs, and multiple routes of administration—intravenous, intra-arterial (carotid artery or via medium cerebral artery catheter), intracerebral and intrathecal—generally leading to improvement in clinical outcome but also frequently associated with adverse events [28]. MSCs have also been used for their immunosuppressive effects in various conditions such as autoimmune diseases or graft-versus-host disease [29]. Part of their beneficial effects are deemed to result from the presence of regulatory T cells (T_reg_) in the preparations [30], known to modulate excessive immune responses [31]. The therapeutic effects of MSC suspensions administered via different routes have been tested in different animal models of MS, generally with positive results [32]. Similar attempts have been made in clinical trials on MS patients [33,34,35,36].

EVs are membrane-enclosed particles released by cells that mediate intercellular communication through the transfer of biologically active molecular species including microRNAs (miRNAs or miRs), proteins, and lipids [37,38]. EVs include several types of particles differing in size and mechanism of generation: exomeres (<50 nm), exosomes (50–150 nm), microvesicles (100–1000 nm), and apoptotic bodies (100–5000 nm) [39,40]. MSC-derived EVs are the most extensively investigated because they may reproduce several beneficial paracrine effects of parental MSCs while avoiding some limitations associated with cell transplantation [39,40,41,42]. Experimental studies in MS models have reported reductions in neuroinflammation and demyelination and enhancement of remyelination following EV administration [41,42]. Experimental EV therapy in other diseases different from MS has been tested in a few clinical studies [39]. More recently, strategies aimed at increasing the therapeutic efficacy of EVs have included preconditioning or genetic modification of parental cells, enrichment of EVs with selected molecular cargo, and surface engineering to improve tissue targeting and biological activity [43,44,45,46,47,48]. Such enhanced/engineered EV preparations represent a developing direction in EV-based therapies for MS.

Previous systematic reviews [39,42] and meta-analyses [41,49] have supported the therapeutic potential of stem cell-derived EVs in preclinical MS models. However, recent studies have expanded this field beyond MSC-derived EVs to include vesicles from other cellular sources, such as oligodendrocytes and oligodendrocyte precursor cells, as well as enhanced/engineered EV preparations. The present meta-analysis provides an updated and broader assessment of the subject, including newer studies and comparisons between native and enhanced/engineered EVs. It also extends the focus of previous meta-analyses, including as outcomes not only clinical or pathological scores, but also different markers of inflammation and remyelination.

## 2. Materials and Methods

The selection of studies to be included in the meta-analysis was conducted in a stepwise manner (identification, screening, eligibility check, inclusion) according to the updated PRISMA guidelines (Preferred Reporting Items for Systematic Reviews and Meta-Analyses) [50,51]. The protocol was registered in the PROSPERO database (CRD420261490822).

### 2.1. Search Strategy

A systematic search of the literature was performed by screening three databases. The PubMed database was interrogated on 4 January 2026 with the following keyword combination: ((stem cell AND exosome OR EVs) AND (multiple sclerosis OR EAE)) NOT (review[Publication Type]).

The results of this initial search were supplemented with papers identified by contiguity in the lists of “Similar articles” or “Cited by” provided by PubMed when accessing each individual entry. The keyword combinations for other literature databases that we searched were as follows.

Web of Science (WoS) was interrogated on 18 November 2025 with the following keyword combination: ((ALL = stem cell AND ALL = exosome) OR ALL = EVs) AND (ALL = multiple sclerosis OR ALL = EAE) NOT DT = Review.

Scopus was interrogated on 4 November 2025 with the following keyword combination: (TITLE-ABS-KEY (stem cell) AND TITLE-ABS-KEY (exosome) OR TITLE-ABS-KEY (EVs)) AND (TITLE-ABS-KEY (multiple sclerosis) OR TITLE-ABS-KEY (EAE)) NOT DOCTYPE (review)).

No language restrictions or exclusion of the gray-zone literature were applied within these searches.

### 2.2. Study Selection

A preliminary assessment of the retrieved publications was performed by analyzing the title and abstract of each entry, followed by a complete analysis, according to the following criteria:

*Inclusion criteria*: (i) original articles; (ii) studies of the impact of EVs, either as primary preparations (native EVs) or with enriched properties (enhanced EVs); (iii) assessment via in vivo administration in rodent animal models of MS; and (iv) presence of quantitative data concerning the modulatory effects of the administered EVs.

*Exclusion criteria*:

− Studies evaluating prophylactic EV administration prior to induction of MS in the animal model;
− Studies assessing effects of in vitro administration of EVs to cells or tissues extracted from MS animal models;
− Studies assessing only immunophenotype markers in cell populations by immunohistochemistry (IHC);
− Studies assessing populations of immune cells in organs other than the CNS (such as spleen or lymph nodes);
− Studies lacking a positive control group;
− Studies that did not report variability measures of quantitative data;
− Studies lacking transparency concerning the number of animals used;
− Studies on animal models of MS using species other than small laboratory rodents (rat or mouse).

Discrepancies in opinions between primary investigators concerning inclusion or exclusion of a study were resolved by a third member of the team. All the publications fulfilling the complete set of inclusion criteria and lacking all exclusion criteria were selected for quantitative assessment.

### 2.3. Data Extraction

Extracted data included measurements related to therapeutic effects of EVs administered in MS animal models (clinical score, pathological score: inflammation, demyelination), measures of myelin repair (oligodendrocyte progenitor cells—OPCs—or oligodendrocyte differentiation markers: PDGFRα, Olig2, PLP, expression level of myelin proteins such as myelin basic protein—MBP—or quantitative assessment of myelin via Black-Gold myelin staining in corpus callosum), and markers of inflammation (TNFα, IFNγ, interleukins) assessed via quantitative RT-PCR, ELISA, Western blotting or IHC. The mean values and measures of variance were extracted in duplicate by two independent assessors. If data were not provided or readily available from the text, they were assessed by manual measurement of the figures and secondarily validated using a certified tool, WebPlotDigitizer v5 (https://automeris.io, accessed between November 2025 and March 2026). Data regarding details of the MS model (strain, group size, age, sex), EV origin, and EV administration (route, dose, frequency, duration of treatment) were also retrieved from each study. If EVs were administered more than once, the results following the last dose were measured in comparison to the control condition. If a variable was measured at multiple time points, the most extreme value was chosen. Clinical scores assessed on scales different than 0–5 (e.g., 0–7) were normalized to the 0–5 scale (multiplying the score by 5/7). For pathological scores of inflammation or demyelination, most studies used 0–3 scales; if different scales (e.g., 0–5) were used or percentages of demyelinated areas were reported, data were normalized proportionally to the 0–3 scale. When a variable was assessed in various studies using different techniques (e.g., myelin basic protein measured by RT-PCR, Western blotting and immunohistochemistry) yielding largely different values, comparison of groups via mean differences (MDs) was replaced by the method of standardized mean differences (SMDs) to account for differences in measurement scales [52,53]. A SMD can be computed starting from the mean difference between treatment and control groups (MD between groups) by dividing it by the pooled standard deviation of the outcome among participants of both groups (*SD*_pooled_), representing the between-participant variability in outcome measurements:

$$\mathrm{SMD}=\frac{\mathrm{difference in mean outcomes between groups}}{\mathrm{standard deviation of outcome among participants}}=$$

$$=\frac{{\mathrm{Mean}\;\mathrm{score}}_{\mathrm{tratment}}-{\mathrm{Mean}\;\mathrm{score}}_{\mathrm{control}}}{{\mathrm{SD}}_{\mathrm{pooled}}}$$

where ${\mathrm{SD}}_{\mathrm{pooled}}=\sqrt{\frac{{\mathrm{SD}}_{\mathrm{treatment}\;}^{2}\left(n_{t}-1\right)+{\mathrm{SD}}_{\mathrm{control}\;}^{2}\left(n_{c}-1\right)}{n_{t}+n_{c}-2}}$ [54,55].

### 2.4. Statistical Analysis

We performed the meta-analysis using Review Manager version 5.4.1 (RevMan), developed by the Cochrane Collaboration [56]. The meta-analysis was performed on continuous outcome data using an inverse-variance method to combine studies, under a DerSimonian and Laird random effects model to account for group and individual variability, with assessment of heterogeneity variance (*τ*^2^), inconsistency (*I*^2^) and 95% confidence intervals. To report treatment effects, we built forest plots. For assessment of publication bias we used funnel plots for comparisons of native EVs vs. enhanced/engineered EVs regarding clinical scores (Figure S2), pathological scores of inflammation (Figure S3) and pathological scores of demyelination (Figure S4). We performed a selection model sensitivity analysis for publication bias with the Meta-Analysis module of JASP v 0.98.1. using *p*-value cut-offs of 0.025 and 0.975 as suggested in [57] to give more weight to observed studies with point estimates in the unexpected direction or with “nonsignificant” *p*-values that are underrepresented in the sample due to publication bias. We also performed a risk of experimental and reporting bias check using the standard criteria included in the SYRCLE RoB tool (Figure S5) [58].

## 3. Results

### 3.1. Selection and General Features of Studies

**Figure 1:**
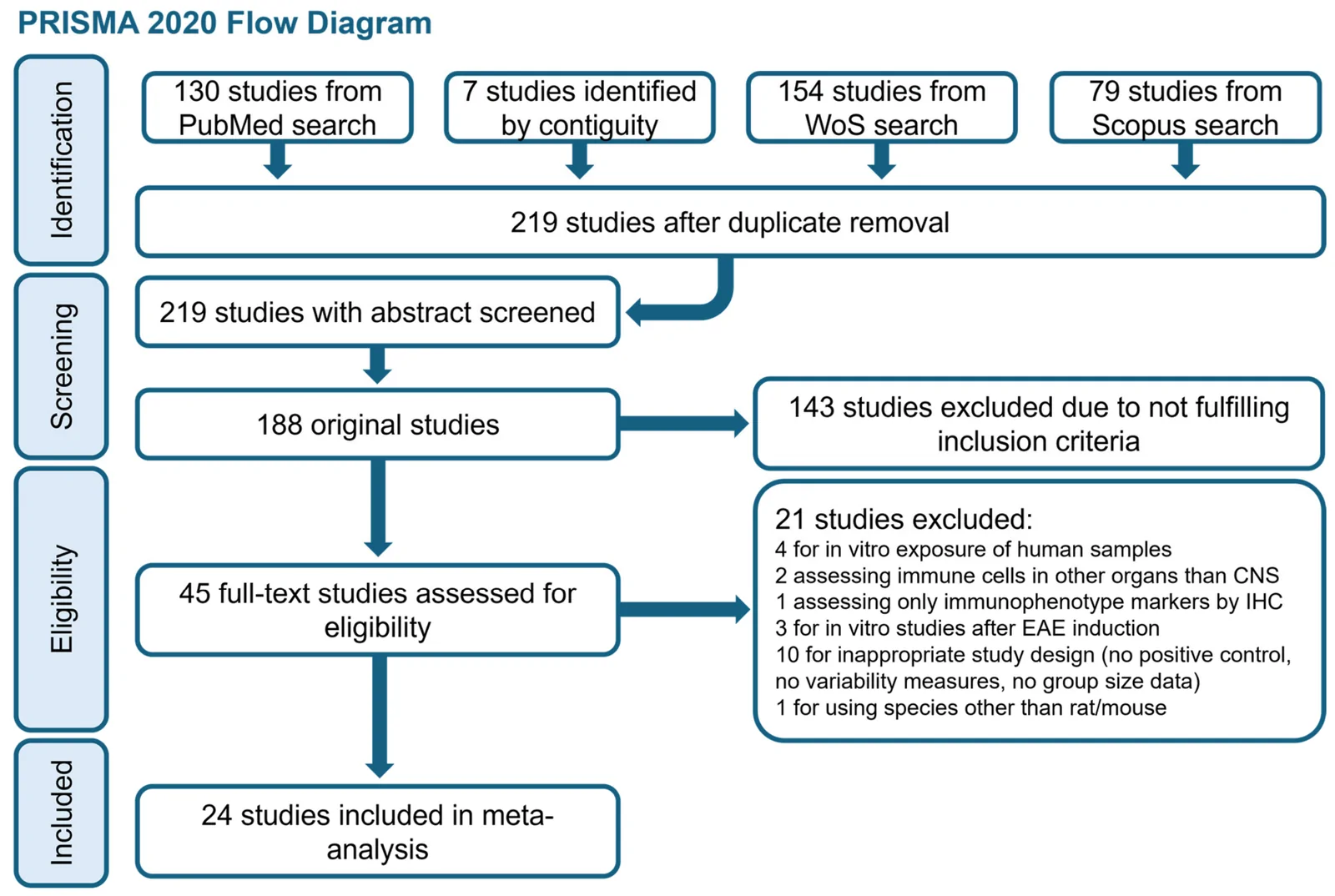
PRISMA flowchart showing the number of studies retrieved and excluded during the identification, initial screening and eligibility check.

**Figure 2:**
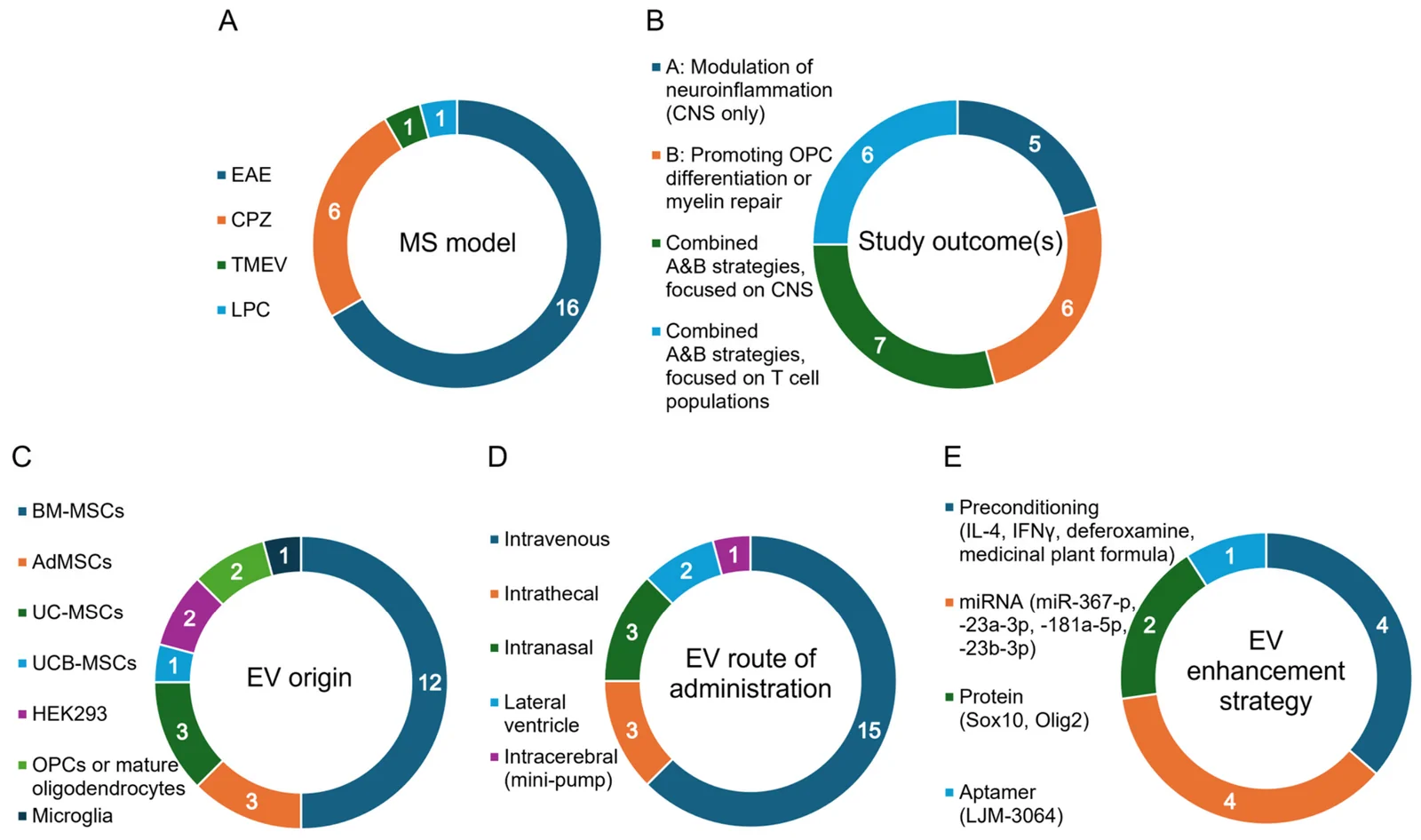
Number of studies included in the meta-analysis classified by the type of MS model (**A**), main study outcomes (**B**), EV origin (**C**), administration route (**D**), and enhancement strategies used in the studies (**E**). Abbreviations: EAE—experimental autoimmune encephalomyelitis; TMEV—Theiler’s murine encephalomyelitis virus; LPC—lysophosphatidylcholine; BM-MSCs—bone marrow mesenchymal stem cells; AdMSCs—adipose tissue mesenchymal stem cells; UC-MSCs—umbilical cord mesenchymal stem cells; UCB-MSCs—umbilical cord blood plasma mesenchymal stem cells; HEK293—human embryonic kidney 293 cells; OPCs—oligodendrocyte progenitor cells; CNS—central nervous system.

By applying the search strategy described in the Section 2 we succeeded in identifying 130 studies in the PubMed database, 154 studies in the WoS database, and 79 studies in the Scopus database with the above-mentioned keyword combinations, while 7 additional studies were retrieved by contiguity from PubMed (Figure 1). Of these, 219 studies were removed upon duplicate search and 143 studies were excluded during preliminary abstract screening for non-compliance with the inclusion criteria. Another 21 studies were excluded during the detailed eligibility checks for various reasons, leaving a total of 24 studies included in the meta-analysis [43,44,45,46,47,48,59,60,61,62,63,64,65,66,67,68,69,70,71,72,73,74,75,76]. Figure 2 illustrates some general features of the studies included in this meta-analysis: the types of MS model, cells of origin for EV preparations, routes of administration of EV preparations, supplementary EV enhancement methods applied in some studies, such as use of cell preconditioning media or EV enrichment with specific miRNAs, proteins or aptamers, and main study outcomes. Figure S1 of the Supplementary Materials visually represents the distribution of techniques used across all the included studies to quantify molecular markers of inflammation and remyelination. Some features of the studies included in this meta-analysis are listed in Table 1: details concerning the MS model and experimental groups (species/strain, number of animals per group, sex, age), EV origin, and EV characterization methods and administration details (route, dose, frequency, duration of therapy), as well as quantitative outcomes assessed in each study grouped by category—clinical/pathological score and inflammation and remyelination markers. Table 2 presents the strategies of EV preparation enhancement used in some of the selected studies.

### 3.2. Clinical and Pathological Scores

**Figure 3:**
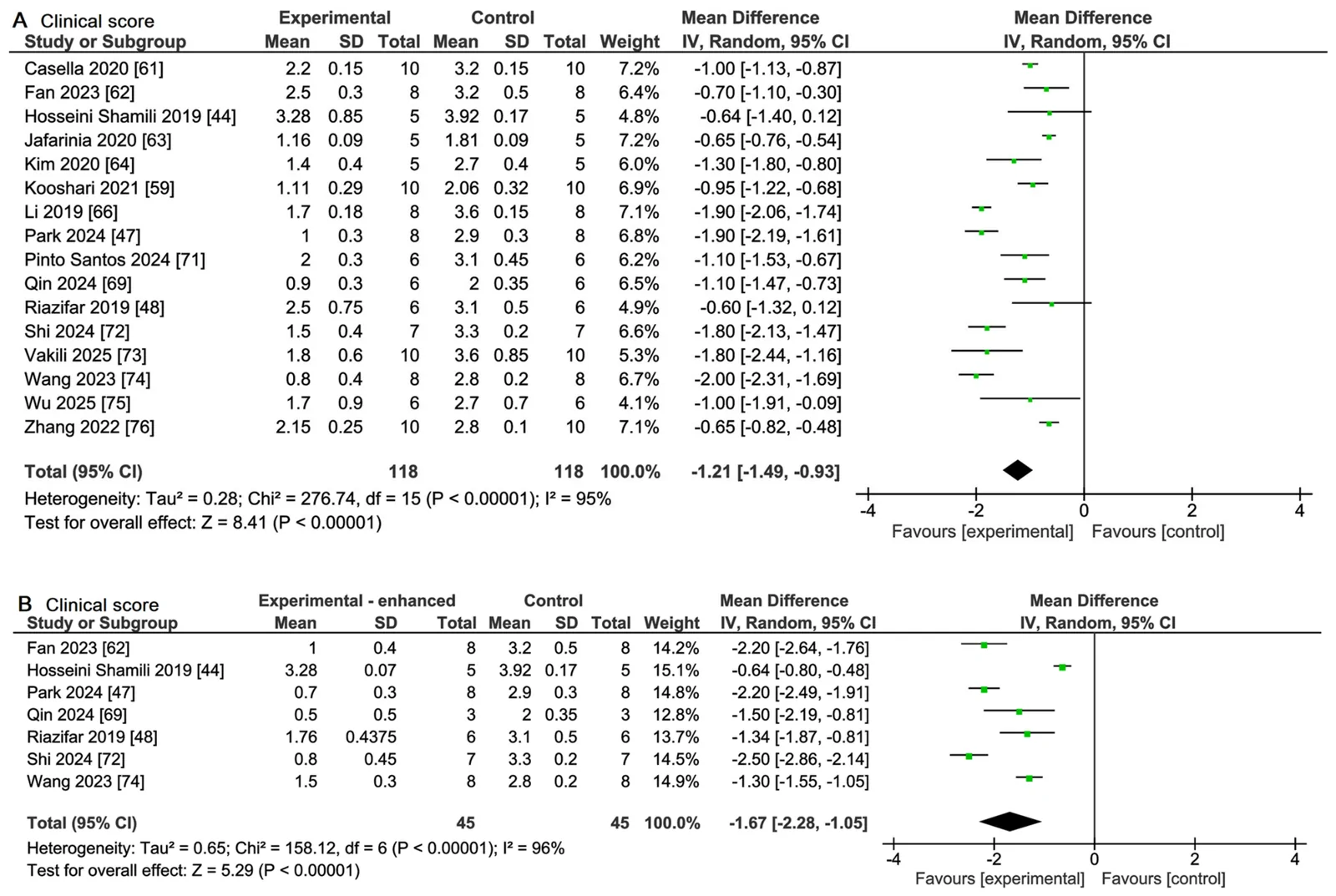
Forest plots of clinical scores for studies using native (**A**) and enhanced (**B**) EV preparations (all scores were scaled to a 0 to 5 interval of values). Differences in clinical score assessment between animals are reported as a mean difference (MD) [44,47,48,59,61,62,63,64,66,69,71,72,73,74,75,76].

The comparison of clinical scores is illustrated in Figure 3. The clinical score grading system is the most common scale used to report daily changes in disability induced specifically by the EAE model, ranging from 0 (healthy animal) to 5 or 7 as maximum values of the scale (exitus). The average effect of treatment with native EVs was a mean difference (MD) of −1.21, with a 95% confidence interval (CI) between [−1.49,−0.93] (Figure 3A), and was even better for enhanced EVs: MD −1.67, CI [−2.28,−1.05] (Figure 3B). Both effects were statistically significant and favored treatment groups relative to control groups in all studies.

**Figure 4:**
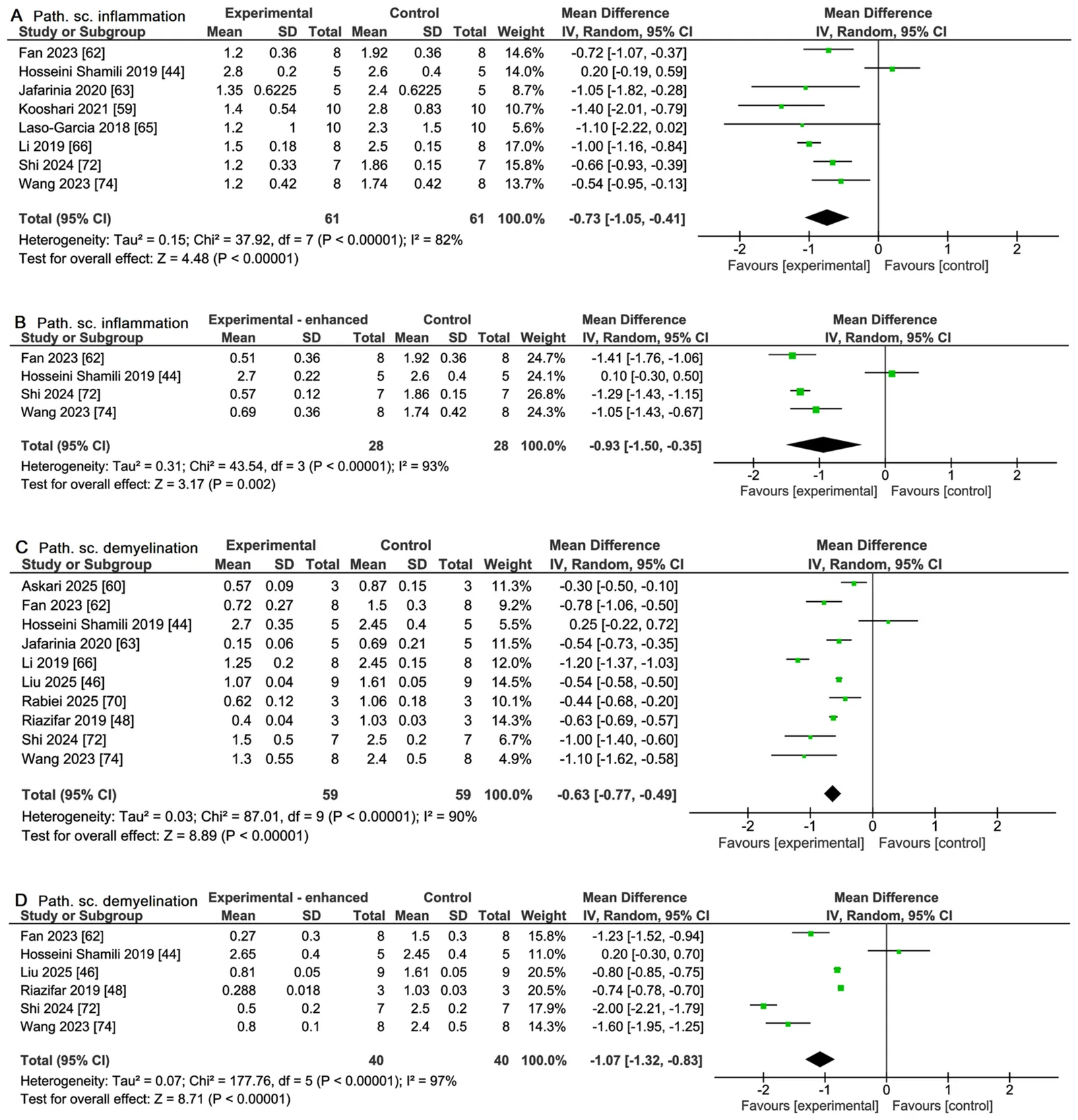
Forest plots of pathological scores of inflammation (**A**,**B**) and demyelination (**C**,**D**) for studies using native (**A**,**C**) and enhanced (**B**,**D**) EV preparations (all scores were scaled to a 0 to 3 interval of values). Differences in pathological score assessment within the CNS (inflammation and demyelination) between histological sections are reported as a mean difference (MD) [44,46,48,59,60,62,63,65,66,70,72,74].

Figure 4 illustrates comparisons of pathological scores of inflammation (Figure 4A,B) and demyelination (Figure 4C,D) for assessment of native and enhanced EV preparations. The pathological score for inflammation was assessed via H&E staining to determine immune cell infiltration into the CNS, conducted on brain or spinal cord sections obtained from EAE and TMEV animal models. Additionally, the pathological score for demyelination was assessed via LFB staining or FluoroMyelin staining of corpus callosum or spinal cord sections obtained from EAE and CPZ animal models. The results again prove improved outcomes in the experimental vs. control groups, and stronger healing effects of enhanced vs. native EV preparations. All results are statistically significant, and the post-treatment improvements in inflammation are higher than the improvements in demyelination scores for native EVs.

### 3.3. Markers of Inflammation

**Figure 5:**
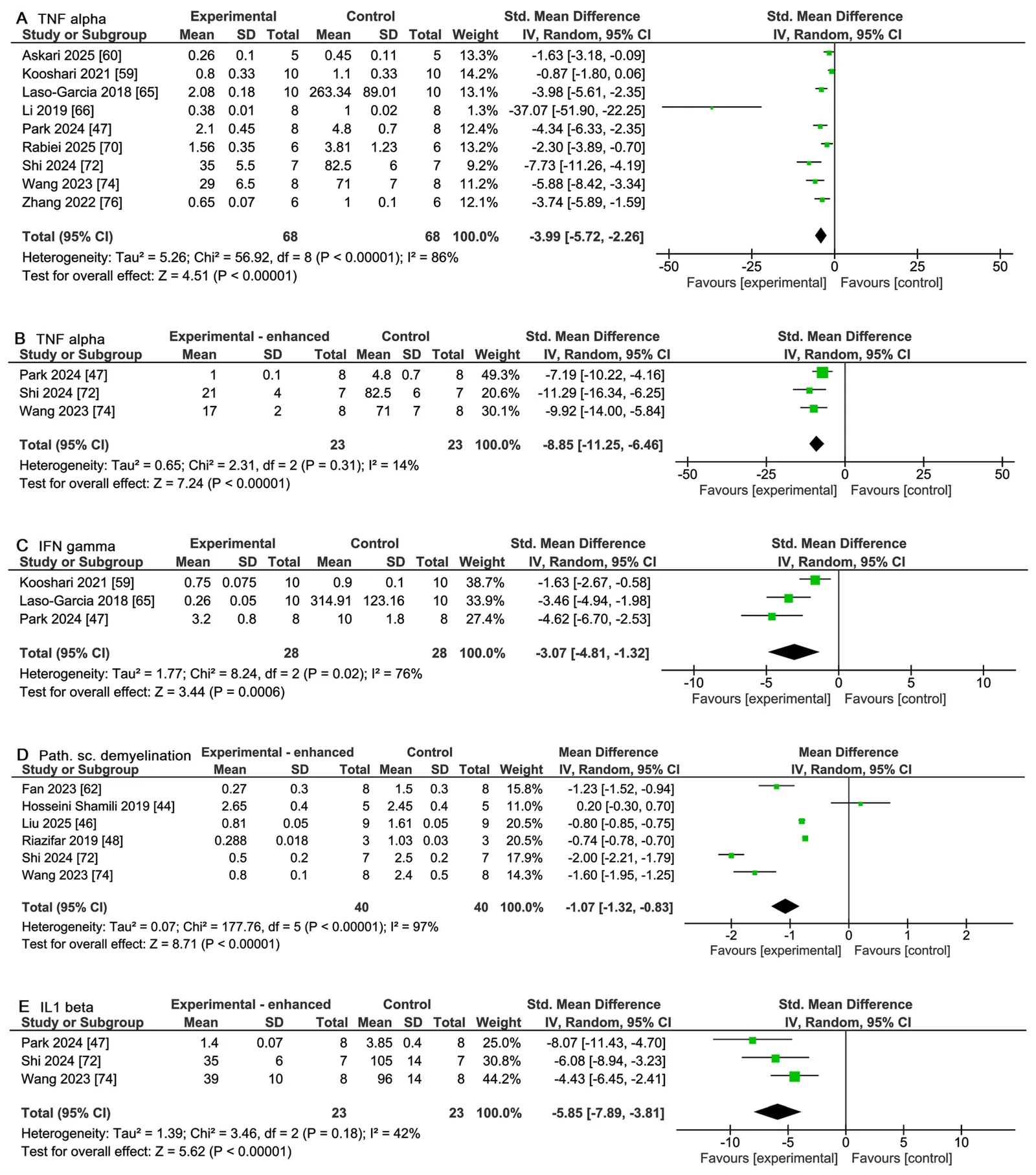
Forest plots of pro-inflammatory cytokines for studies using native (**A**,**C**,**D**) and enhanced (**B**,**E**) EV preparations. Differences in neuroinflammation assessment between the quantified level of each inflammation marker are represented as a standardized mean difference (SMD) [44,46,47,48,59,60,62,65,66,70,72,74,76].

**Figure 6:**
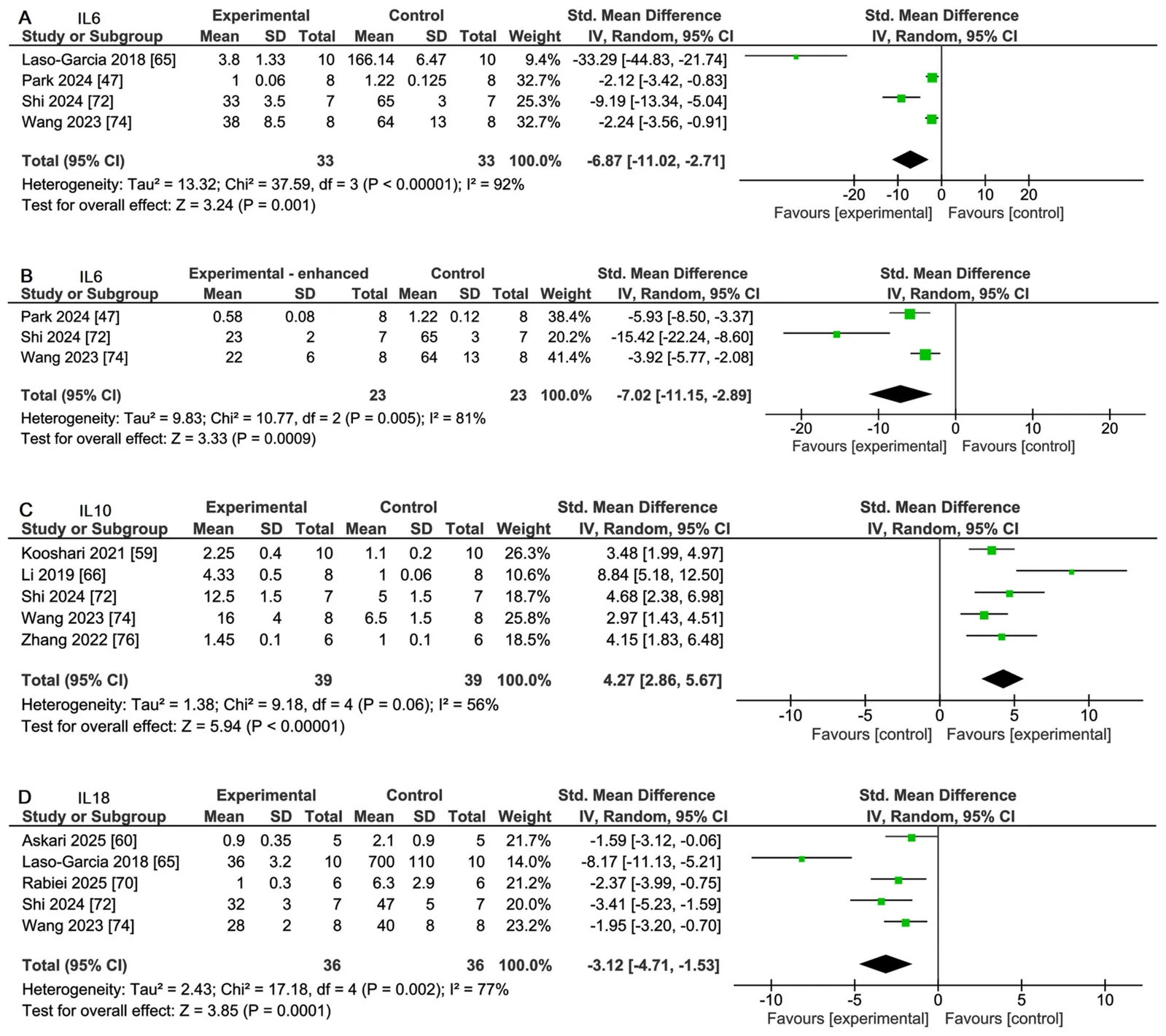
Forest plots of pro-inflammatory (**A**,**B**,**D**) and anti-inflammatory (**C**) cytokines for studies using native (**A**,**C**,**D**) and enhanced (**B**) EV preparations. Differences in neuroinflammation assessment between the quantified level of each inflammation marker are represented as a standardized mean difference (SMD) [47,59,60,65,66,70,72,74,76].

The meta-analysis included comparisons between experimental and control groups for several general markers of inflammation assessed in nervous tissue or blood plasma/serum at the mRNA or protein level: TNFα (Figure 5A,B), IFNγ (Figure 5C), IL-1β Figure 5D,E), IL-6 (Figure 6A,B), IL-10 (Figure 6C), and IL-18 (Figure 6D). Due to inter-study differences in experimental methods, comparisons were performed using standardized mean differences (SMDs). For all inflammation markers we found statistically significant differences between groups for native EVs and even greater differences for enhanced EVs. A comprehensive list of EV effects in modulating inflammation is presented in Table S1 (upper section).

### 3.4. Markers of Remyelination

**Figure 7:**
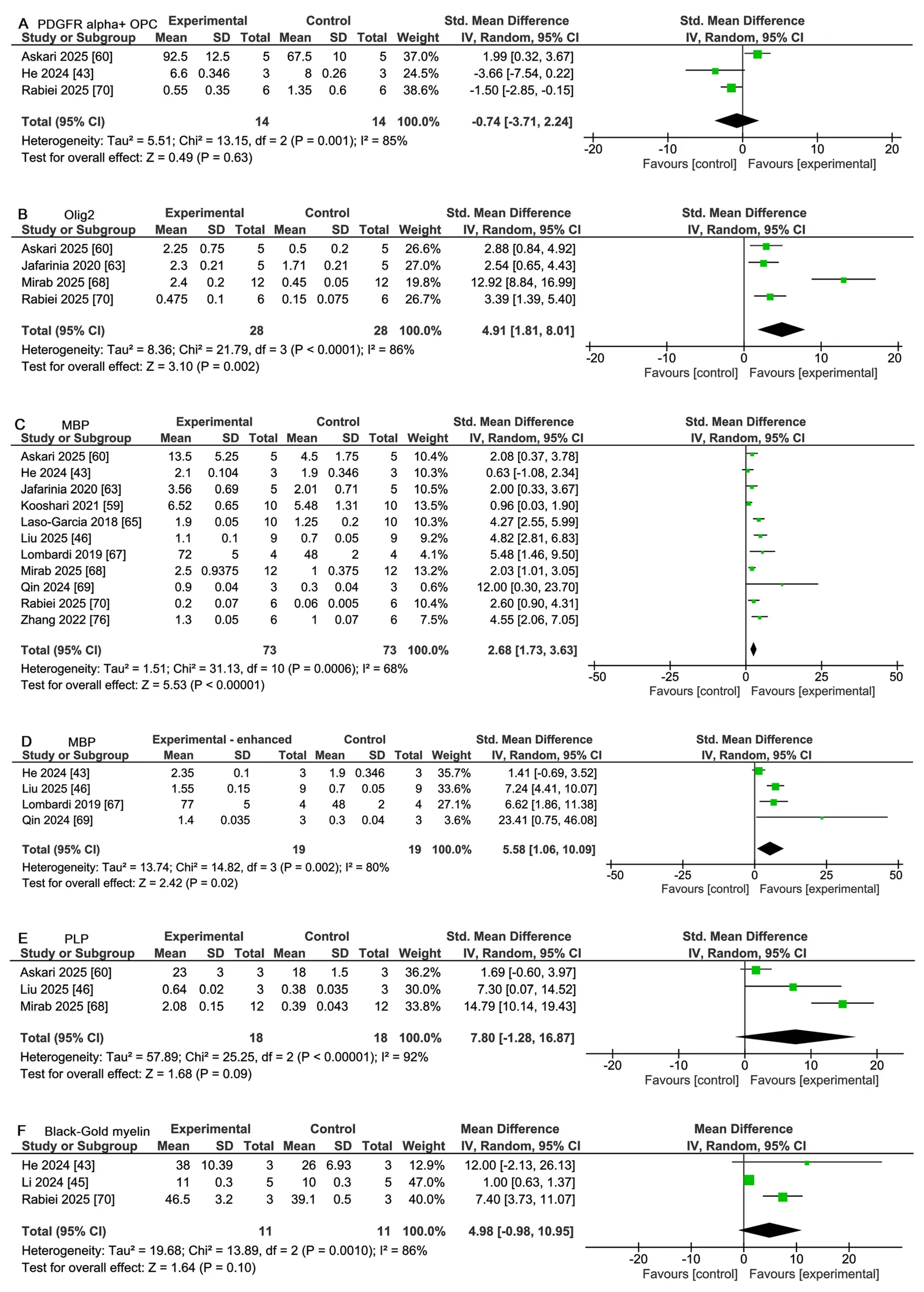
Forest plots of myelin repair markers through different maturation stages of oligodendrocytes (**A**–**E**), as well as histological markers of myelin via Black-Gold staining (**F**) for studies using native (**A**,**B**,**C**,**E**,**F**) and enhanced (**D**) EV preparations. Differences in remyelination assessment for the quantified level of each myelination marker are represented as a standardized mean difference (SMD) [43,45,46,59,60,63,65,67,68,69,70,76].

We also assessed differences between experimental groups concerning several markers of remyelination, including oligodendrocyte or progenitor (OPC) markers, PDGF receptor α (Figure 7A) and Olig2 (Figure 7B); myelin protein markers such as myelin basic protein (MBP) (Figure 7C,D) and proteolipid protein (PLP) (Figure 7E); and myelin content of white matter tracts (generally corpus callosum), quantified using Black-Gold myelin staining (Figure 7F). For all these markers, except for PDGFRα, there were statistically significant differences between groups favoring the EV therapy groups. Again, the enhanced EV preparations performed better than native EVs for MBP, leading to standardized mean differences (SMDs) and 95%CIs of 2.68 [1.73,3.63] for native EVs (Figure 7C) vs. 5.58 [1.06,10.09] for enhanced EVs (Figure 7D). A comprehensive list of EV effects in modulating remyelination is presented in Table S1 (lower section).

### 3.5. Risk of Experimental and Publication Bias Analyses

We performed several supplementary analyses in order to assess the risk of experimental, reporting and publication bias. First, we performed a risk of experimental and reporting bias assessment using the standard criteria defined in the SYRCLE RoB tool. As shown in Figure S5 of the Supplementary Materials, these criteria address selection bias (random allocation sequence generation, group adjustment for confounders, adequate allocation of animals in experimental groups), performance bias (random housing, blinding of caregivers/investigators), detection bias (random animal selection for outcome assessment, assessor blinding), attrition bias (incomplete outcome data), selective reporting bias, and other types of bias. Problems were identified in group adjustment for confounders and attrition bias. We also performed a selection model sensitivity analysis for publication bias with the Meta-Analysis module of JASP v 0.98.1, using *p*-value cut-offs of 0.025 and 0.975. The publication bias for each analyzed outcome resulted in adjusted values of effects using a random effects and, when computable, also a fixed effects hypothesis. The adjusted values and 95% confidence intervals are reported in tables and graphs in Figures S6–S26 of the Supplementary Materials.

## 4. Discussion

This meta-analysis of 24 publications shows that in animal models of MS, stem-cell- and glial-cell-released EVs demonstrate great therapeutic potential. Both the native and the function-enhanced EVs alleviated disease severity in preclinical MS models, assessed through the standardized clinical scoring system or CNS pathological scores for inflammation and demyelination. These findings were highly significant and correlated with the improved interplay between neuroinflammation and remyelination, as interpreted through cytokine dosing and via quantitative expression of myelin proteins and OPC differentiation markers.

Even though MS is unique to humans, extensive research in animal models has tried to provide translatable insights regarding the disease. However, the majority of MS animal models, including the EAE and Cuprizone models, fail to fully recapitulate the pathophysiological features of MS [77]. EV treatments showed promising clinical and pathological scores when EAE was induced in C57BL/6 mice in 15 out of the 16 selected EAE studies. However, the study of relapses seems elusive in the C57BL/6 EAE MS model [10]. While EAE provides no insights into MS progression [78], only one study used TMEV inoculation to assess both the progressive stages and viral hypothesis of the disease [79,80]. All seven studies that used toxic demyelination models successfully addressed myelin repair mechanisms induced by EV treatment in spatiotemporally predictable (LPC) or unpredictable (CPZ) lesions; however, these models promote inherent accelerated remyelination with no regard to progressive MS remyelination failure [81], or to the complexity of OPC recruitment and differentiation mechanisms [82]. To summarize, none of the MS animal models are perfect. EAE via MOG 35–55 injection incorporates features related to both inflammation and demyelination, and should be preferred for assessment of therapeutic effects on the microglial component, a key target of EV therapy [39], while the Cuprizone model primarily reproduces remyelination phenomena and is sensitive to effects on the OPC/oligodendrocyte components. Both models are relatively easy to implement, while the TMEV model, although more realistic, requires viral manipulation, intracranial inoculation and strict follow-up [65,83]. Despite differences largely related to the lesions, such as their topography, morphopathology, and stages of evolution, all these animal models feature the essential pathophysiological features present in MS—inflammation, demyelination and remyelination—and can be combined for assessing quantitative markers that characterize the pathophysiology of MS, like in the current meta-analysis or the previous similar study by Barabadi et al. [41].

The studies selected for meta-analysis show a surprising variety of sources for EV preparations. A majority of studies used cell preparations of mesenchymal stem cell origin, such as BM-MSCs, UC-MSCs, UCB-MSCs, and AdMSCs; only two studies used mouse primary OPCs and oligodendrocytes, one study used rat primary microglia, and the other two studies used HEK293 cells, out of which microglia- and HEK293-cell-derived EVs were specifically subjected to enhancement strategies to improve their therapeutic properties. The MSC-derived EV preparations were obtained from cells of human origin (7 of the 19 remaining studies) or animal origin: mouse (seven studies), rat (three studies), dog (one study) and rhesus monkey (one study). Generally, EV preparations of MSC origin produced better results in terms of both inflammation and remyelination markers when compared to cells from other origins; native EVs from HEK293 cells yielded the least favorable results, and may be used as negative controls. These striking differences may be explained by the rich molecular content of MSC EVs, particularly the miRNA species with beneficial effects, including miR134 promoting oligodendrocyte survival [84], miR146a-5p and miR125a modulating the reactive microglia phenotype [85,86]. This microglial conversion is deemed to result from modulation of several innate or adaptive immunity signaling pathways involving molecular players like TLR4, PI3K/AKT, NF-κB, and others [87]. A recent study links focal white matter lesions to gray matter microgliosis and synapse engulfment, required downstream for white matter remyelination [88]. Shedding light onto the need for multi-dimensional therapeutic approaches that balance both inflammation and remyelination in MS [89,90], EV therapy in preclinical MS models has already begun to address this connection; such an approach is present in 13 out of the 24 included studies. Regarding the remyelination component of the meta-analysis, the decrease in PDGFRα is neither elusive, nor accidental. After EV administration, this early-stage marker snapshots the transition of the immature OPC pool (first stage), rich in PDGFRα, towards the pre-oligodendrocyte (or late OPC) stage of progression [91,92], thus losing most of PDGFRα expression. During this transition, other markers such as Olig2 increase according to their specific expression in both the late OPC stage (second stage) and immature oligodendrocyte stage (third stage) [93]. In such a continuum, other mature oligodendrocyte-specific markers, MBP and PLP, present in late, myelinating oligodendrocytes (fourth stage) [91] and are marked by increased expression levels after EV treatment. To support the effects found at the molecular level in oligodendrocyte lineage markers, assessing myelin integrity via Black-Gold staining histologically confirms the transition towards myelin restoration within the corpus callosum in three studies employing the CPZ model.

The 24 studies selected for meta-analysis generally provided an accurate description of EV preparation methods and an in-depth characterization of their size and properties. Different concurrent methods for size measurement were used, including various electron microscopy techniques (TEM, SEM, Cryo-EM), atomic force microscopy (AFM), light scattering and zeta potential measurements (dynamic light scattering—DLS; nanoparticle tracking analysis—NTA). EV diameters follow mono- or multi-Gaussian distributions, with mean diameters of the main component below 150 nm or more often below 100 nm (Table 1). Given the size, they are more prone to traverse the BBB and enter injured CNS tissue than MSCs themselves. Furthermore, EV content was characterized with multiple methods, including detection of specific protein markers such as MSC-specific CD (cluster of differentiation) antigens by Western blotting (WB) or dot-blotting, flow cytometry, and fluorescence-activated cell sorting (FACS). Spectroscopy methods, as well as liquid chromatography–mass spectrometry (LC-MSpec), sometimes preceded by enzyme digestion such as in shotgun proteomics assays, were rarely employed in EV surface marker detection. Few studies attempted a detailed characterization of the molecular content of the EVs using transcriptomics methods like RT-qPCR (reverse transcription–quantitative polymerase chain reaction) or RNAseq (RNA deep sequencing). The sphingolipid content of the EVs was measured in a single study. It is worth mentioning that quality assessment of EV preparations and standardized production methods ensuring a uniform quality are essential for clinical translatability. EV preparation and assessment methods should observe the recommendations issued by the International Society for Extracellular Vesicles (ISEV), namely the most recent version of Minimal Information for Studies of Extracellular Vesicles (MISEV) guidelines (https://www.isev.org/misev (accessed on 30 September 2026)) [94,95,96]. Given that the standards and assays tend to become more stringent and complex, it may be of interest for future studies to externalize EV quality assessment to specialized companies with expertise in this field, e.g., Creative Biolabs or System Biosciences, an approach already taken in one of the studies [64].

Another important issue is related to the route of administration, dose, and frequency of application. Since the EV preparations were highly concentrated (on average 10^10^ EVs per ml), the injected amounts were generally between 10 and 100 μg per dose—in a few studies 150 μg and in a single study 400 μg—and the administration route was intravenous in most studies, via the tail vein; in a few studies it was intracerebroventricular, intrathecal, intranasal or continuous via an intracerebral mini-pump (Figure 2 and Table 1). The frequency and duration of applications varied from a single dose to three or more doses over time intervals lasting from one week to four weeks. For clinical translatability, beyond the convenient intravenous route, other routes such as intra-arterial internal carotid catheters (due to efficacy) or the intranasal route (due to reduced invasiveness) may be attractive.

A main result of our meta-analysis is the improved effect of enhanced EV preparations vs. native EVs on different outcomes such as clinical and pathological scores (both demyelination and inflammation) as well as specific markers of inflammation and remyelination. Since all studies reported better results with enhanced than with native EVs, it remains an open question whether studies showing opposite effects (better for native than enhanced EVs) tended to be underreported. Another sensitive point is the possible bias in assessment in order to present groundbreaking improvements in quality with enhanced EVs. In any case, native EVs themselves proved reasonable levels of effectiveness in most studies, thus representing an attractive, cheaper and more affordable option for clinical application. The methods used for EV enhancement were extremely diverse (Table 2 and Figure 2), ranging from supplementation with exogenous miR species (4 of 10 studies) or proteins (one study), to aptamer conjugation (one study) or preconditioning of cells or animals of origin (four studies). Thus, it is impossible to select an optimal method or protocol for EV enhancement, and the outcome in the case of combination of different enhancement methods (e.g., multiple exogenous miRNA species) remains uncertain due to possible antagonistic effects. Moreover, the increased specificity of therapeutic oligonucleotide-based strategies—such as aptamer LJM-3064 that binds to myelin and rapidly distributes within the CNS, promoting remyelination [97]—failed to provide exceptional results when conjugated with BM-MSC-derived EVs [44]. However, similar therapeutic strategies that use even more refined aptamers such as LJM-5708 might still have potential to be tested in preclinical models when conjugated with EVs.

Another open question is related to a comparison of effectiveness between EV therapy and other groundbreaking clinical therapies proposed recently for MS, such as engineered Treg preparations. Chimeric antigen receptor (CAR)-T cell therapy might encompass promising disease-specific treatment effects for autoimmune neurological disorders [98]. While in MS murine models fine-tuned MOG CAR regulatory T (Treg) cells were shown to bind to oligodendrocytes and protect them against immune attack in both pre-induced and secondary challenged EAE [99], further human studies with larger MS patient cohorts are needed for CAR T cell therapy to be critically validated in progressive MS [100]. Another appealing option, already in clinical use, is represented by CD20 monoclonal antibodies such as rituximab, ocrelizumab and ofatumumab, which reduce populations of CD4+ T cells and B cells reactive to Epstein–Barr virus late lytic antigens, considered important for the etiopathogenesis of MS [7].

Our meta-analysis has a number of limitations. Heterogeneity indicators such as inconsistency *I*^2^, between-study variance *τ*^2^, and *p*-value of *Q* statistic, introduced by Cochran in 1954 [101], demonstrate high levels of inter-study heterogeneity [102]. For this reason, we performed the statistical analyses with a DerSimonian and Laird random effects model instead of a fixed effects model. For inter-study comparisons for markers that were assessed with different methods in different studies (e.g., RT-qPCR, WB, ELISA, etc., as shown in Figure S1 of the Supplementary Materials), due to large variations in the reported values, we preferred to use the standardized mean difference (SMD) instead of the mean difference (MD). However, the reliability of such comparisons is limited, and there is no direct equivalence between quantitative expression at the mRNA and protein levels. Another limitation is the lack of focus given to animals that did not survive to the end of the study; generally, they were not reported (except for inclusion in clinical scores), and obviously they can influence the final outcomes. Other limitations of our meta-analysis include possible selective reporting, incomplete EV characterization, small sample sizes, heterogeneity across MS models, a lack of long-term outcomes, and the absence of standardized functional endpoints. To address the problem of publication bias we performed supplementary analyses: funnel plots, selection model sensitivity analysis, and risk of experimental and reporting bias analysis. However, any publication bias analysis method (funnel plots, Egger’s regression asymmetry test, trim-and-fill analysis, p-curve-type approaches) cannot fully account for significant reporting bias. The selection model to assess sensitivity to publication bias, a method that we applied, can identify situations where funnel plots or asymmetry tests do not signal any problem, but, like many statistical methods, it requires arbitrary statistical assumptions, such as the assumption that the true effects are normally distributed, independent, and not correlated with the point estimates’ standard errors [57].

## 5. Conclusions

In conclusion, our meta-analysis succeeded in evidencing statistically significant differences between EV treatment and control groups in clinical scores and several important markers of neuroinflammation and demyelination/remyelination. These differences were higher for enhanced EV preparations than for the equivalent native EV preparations. Thus, MSC EV therapies could provide reliable clinical options. However, the problem of standardization/quality control is crucial. On the long way to clinical translatability, selection of patients is an unsolved problem, as well as other details of therapy regimes (dose, route, duration, etc.). The mechanisms of action are complex and incompletely understood; adverse effects are also largely unknown at this point, possibly including excessive immunosuppression leading to development of malignant diseases or severe infections (e.g., fungal infections). It is also not clear how this therapy could be combined with other therapies for MS. Unlike experiments on animal models, in clinical MS cases medical doctors do not have the luxury of knowing when the disease or the specific exacerbation started, so they need to know how flexible the onset of treatment can be while still being effective and definitely not making the outcome worse. Therefore, future studies will be needed to evaluate the effects of different treatment onset times as well as doses (requiring dose–response curves) on efficacy and potential side effects.

## Figures and Tables

**Table 1:** Synopsis of data extracted from 24 studies included in the meta-analysis assessing EVs’ therapeutic effects in rodent animal models of MS, comprising records of both native EVs and enhanced/engineered EVs.

Article	MS Model; Strain; Group Size; Sex; Age	EV Origin	EV Characterization Methods: Size and Features	EV Administration: Route; Dose; Frequency; Duration of EV Treatment	Quantified Outcome(s) Included in Meta-Analysis
Askari H et al. 2025 [60]	CPZ, C57BL/6 mice, 5/group or 3/group, male, 6 weeks old	Bone marrow mesenchymal stem cells (BM-MSCs) from Sprague–Dawley rats	93 nm average by DLS and TEM, surface markers by WB, protein content by BCA	Intravenous injection, 150 μg, two doses for 14 days, followed by the endpoint	Myelin repair: OPCs or oligodendrocyte differentiation markers and expression of myelin proteins via qRT-PCR (*PDGFRα*, *Olig2*, *MBP*) or IHC (PLP) in the corpus callosum Neuroinflammation: Cytokines via qRT-PCR (*TNFα*, *IL-1β*, *IL-18*) in the corpus callosum Pathological score: Demyelination via IHC (FluoroMyelin staining) in corpus callosum
Casella G et al. 2020 [61]	EAE, C57BL/6 mice, 10/group, n/a, n/a	Oligodendrocyte primary cultures from C57BL/6 mice	240 nm average by Cryo-EM and NTA, surface markers by MSpec and ELISA	Intravenous injection, 1 × 10^10^ particles, three doses at three days interval for 7 days, followed by 12 days until the endpoint	Clinical score: 0–5 scale
Fan J et al. 2023 [62]	EAE, C57BL/6 mice, 8/group, female, 8–10 weeks old	Bone marrow mesenchymal stem cells (BM-MSCs) from C57BL/6 mice	105 nm by TEM and NTA, surface markers by WB	Intrathecal injection, 10 μg, single dose, followed by 7 days until the endpoint	Clinical score: 0–5 scale Pathological score: Inflammation (H&E staining) and demyelination (LFB staining) in the spinal cord
He J et al. 2024 [43]	CPZ, C57BL/6 mice, 3/group, male, 8 weeks old	HEK293 cells	127 nm average by NTA and TEM, surface markers by WB	Lateral ventricle injection, 1 × 10^9^ particles, single dose, followed by 10 days until the endpoint	Myelin repair: OPCs or oligodendrocyte differentiation markers and expression of myelin proteins via IHC (PDGFRα, MBP) in the corpus callosum; quantitative assessment of myelin via IHC (Black-Gold staining, % per field) in the corpus callosum
Hosseini Shamili F et al. 2019 [44]	EAE, C57BL/6 mice, 3–5/group, female, 10–13 weeks old	Bone marrow mesenchymal stem cells (BM-MSCs) from C57BL/6 mice	101 nm by AFM and DLS, surface markers by FACS, WB and BCA	Intravenous (tail vein) injection, 200 μg, three doses at three days interval for 7 days, followed by 10 days until the endpoint	Clinical score: 0–7 scale Pathological score: Inflammation (H&E staining) and demyelination (LFB staining) in the spinal cord
Jafarinia M et al. 2020 [63]	EAE, C57BL/6 mice, 5/group, female, 6–8 weeks old	Adipose mesenchymal stem cells (AdMSCs) from human samples	85 nm average by DLS and TEM, surface markers by FACS and BCA	Intravenous injection, 60 μg, single dose, followed by 20 days until the endpoint	Clinical score: 0–7 scale Pathological score: Inflammation (H&E staining) and demyelination (LFB staining) in the spinal cord Myelin repair: OPCs or oligodendrocyte differentiation markers and expression of myelin proteins via IHC (Olig2, MBP) in the spinal cord
Kim S et al. 2020 [64]	EAE, C57BL/6 mice, 5/group or 3/group, n/a, n/a	Umbilical cord blood plasma cells (UCB-MSCs) from human samples	81 nm average by DLS, TEM and NTA, surface markers by FACS and dot-blot (using an exosomal antibody array kit)	Intravenous injection, 100 μg, one dose every other day for 10 days, followed by 10 days until the endpoint	Clinical score: 0–5 scale
Koohsari S et al. 2021 [59]	EAE, C57BL/6 mice, 10/group, female, 6–8 weeks old	Umbilical cord mesenchymal stem cells (UC-MSCs) from human samples	91 nm average by DLS and SEM, surface markers by FACS	Intravenous injection, 50 μg, single dose, followed by 21 days until the endpoint	Clinical score: 0–7 scale Pathological score: Inflammation (H&E staining) in the spinal cord Myelin repair: Expression of myelin proteins via IHC (MBP) in the spinal cord Neuroinflammation: Cytokines via qRT-PCR (*TNFα*, *IFNγ*, *IL-10*) in the spinal cord
Laso-Garcia F et al. 2018 [65]	TMEV, SJL mice, 10/group, female, 4–6 weeks old	Adipose mesenchymal stem cells (AdMSCs) from human samples	<100 nm by TEM and NTS, surface markers by WB and BCA	Intravenous (tail vein) injection, 25 μg, single dose, followed by 15 days until the endpoint	Pathological score: Inflammation (H&E staining) in the spinal cord Myelin repair: Expression of myelin proteins via WB (MBP) in the brain Neuroinflammation: Cytokines via ELISA (TNFα, IFNγ, IL-1β, IL-6, IL-18) in plasma
Li Y et al. 2024 [45]	CPZ, C57BL/6 mice, 5/group, male, 8 weeks old	HEK293 cells	124 nm average by NTA and TEM, surface markers by WB, EV phagocytosis assay	Lateral ventricle injection, 1 × 10^9^ particles, single dose, followed by 10 days until the endpoint	Myelin repair: Quantitative assessment of myelin via IHC (Black-Gold staining, % per field) in the corpus callosum
Li Z et al. 2019 [66]	EAE, Sprague–Dawley rats, 8/group, female, n/a	Bone marrow mesenchymal stem cells (BM-MSCs) from Sprague–Dawley rats	30–100 nm by TEM, surface markers by WB, shotgun proteomics (LC-MSpec)	Intravenous (tail vein) injection, 400 μg, single dose, followed by 13 days until the endpoint	Clinical score: 0–5 scale Pathological score: Inflammation (H&E staining) and demyelination (LFB staining) in the spinal cord Neuroinflammation: Cytokines via ELISA (TNFα, IL-10) in serum
Liu SS et al. 2025 [46]	CPZ, C57BL/6 mice, 9/group, female, n/a	Bone marrow mesenchymal stem cells (BM-MSCs) from C57BL/6 mice	30–150 nm by NTA and TEM, surface markers by WB and BCA, miRNA sequencing,	Intravenous (tail vein) injection, 10 μg, two doses for 14 days, followed by the endpoint	Myelin repair: Expression of myelin proteins via WB (MBP) or IHC (PLP) in the brain Pathological score: Demyelination (LFB staining) in the corpus callosum
Lombardi M et al. 2019 [67]	LPC, C57BL/6 mice, 3–5/group, male, 10–16 weeks old	Microglia primary cultures from Sprague–Dawley rats	81 nm average by Cryo-EM and NTA, molecular composition by Raman spec, sphingolipid content, cytokine profile via ELISA	Mini-pumps, 2 × 10^8^ particles, intracerebral continuous delivery for 4 days, followed by the endpoint	Myelin repair: Expression of myelin proteins via IHC (MBP) in the brain
Mirab M et al. 2025 [68]	CPZ, C57BL/6 mice, 12/group, male, n/a	Umbilical cord mesenchymal stem cells (UCB-MSCs) from human samples	74 nm average by DLS and TEM, surface markers by WB and BCA	Intravenous (tail vein) injection, 50 μg, one dose per week for 21 days, followed by the endpoint	Myelin repair: OPCs or oligodendrocyte differentiation markers and expression of myelin proteins via qRT-PCR (*Olig2*, *MBP*, *PLP*) in the corpus callosum
Park SM et al. 2024 [47]	EAE, C57BL/6 mice, 8/group, female, 6 weeks old	Adipose tissue mesenchymal stem cells (AdMSCs) from canine samples	100–200 nm by TEM and DLS, surface markers by FACS and WB	Intranasal injection, 10 μg, daily dose for 14 days, followed by 2 days until the endpoint	Clinical score: 0–5 scale Neuroinflammation: cytokines via qRT-PCR (*TNFα*, *IFNγ*, *IL-1β*, *IL-6*) in the spinal cord
Pinto Santos SI et al. 2024 [71]	EAE, C57BL/6 mice, 6/group, female, 10 weeks old	Oligodendrocyte progenitor cells (OPCs) differentiated from C57BL/6 mice neural stem cells	155 nm average by NTA and TEM, surface markers by WB	Intravenous (tail vein) injection, 2.5 × 10^9^ particles, single dose, followed by 15 days until the endpoint	Clinical score: 0–5 scale
Qin D et al. 2024 [69]	EAE, C57BL/6 mice, 3/group or 6/group, male, 6 weeks old	Umbilical cord mesenchymal stem cells (UC-MSCs) from human samples	143 nm average by NTA and TEM, surface markers by WB and BCA	Intravenous injection, 50 μg, single dose, followed by 15 days until the endpoint	Clinical score: 0–5 scale Myelin repair: Expression of myelin proteins via WB (MBP) in the brain
Rabiei F et al. 2025 [70]	CPZ, C57BL/6 mice, 3/group or 6/group, male, 6–8 weeks old	Bone marrow mesenchymal stem cells (BM-MSCs) from Sprague–Dawley rats	93 nm average by DLS and TEM, surface markers by WB and BCA	Intranasal infusion, 10 μg, four doses for 14 days, followed by 2 days until the endpoint	Pathological score: Demyelination via IHC (FluoroMyelin staining) in the corpus callosum Myelin repair: OPCs or oligodendrocyte differentiation markers and expression of myelin proteins via qRT-PCR (*PDGFRα, Olig2, MBP)* in the corpus callosum; quantitative assessment of myelin via IHC (Black-Gold staining, % per field) in the corpus callosum Neuroinflammation: Cytokines via qRT-PCR (*TNFα*, *IL-1β*, *IL-18*) in the corpus callosum
Riazifar M et al. 2019 [48]	EAE, C57BL/6 mice, 3/group or 6/group, female, 6–8 weeks old	Bone marrow mesenchymal stem cells (BM-MSCs) from human samples	115 nm average by NTA and TEM, surface markers by FACS and WB, BCA, LC-MSpec, RT-PCR, RNAseq	Intravenous injection, 150 μg, single dose, followed by 22 days until the endpoint	Clinical score: 0–4 scale * Pathological score: Demyelination (LFB staining) in the spinal cord
Shi Z et al. 2024 [72]	EAE, C57BL/6 mice, 7/group, female, 8–10 weeks old	Bone marrow mesenchymal stem cells (BM-MSCs), n/a	135 nm average by DLS, NTA and TEM, surface markers by WB	Intrathecal injection, 10 μg, single dose, followed by 7 days until the endpoint	Clinical score: 0–5 scale Pathological score: Inflammation (H&E staining) and demyelination (LFB staining) in the spinal cord Neuroinflammation: Cytokines via ELISA (TNFα, IL-1β, IL-6, IL-10, IL-18) in the spinal cord
Vakili S et al. 2025 [73]	EAE, C57BL/6 mice, 10/group, female, 6–8 weeks old	Bone marrow mesenchymal stem cells (BM-MSCs) from C57BL/6 mice	104 nm average by DLS, surface markers by WB	Intravenous injection, 100 μg single dose, followed by 21 days until the endpoint	Clinical score: 0–5 scale
Wang J et al. 2023 [74]	EAE, C57BL/6 mice, 8/group, female, 8–10 weeks old	Bone marrow mesenchymal stem cells (BM-MSCs) from C57BL/6 mice	134 nm average by NTA and TEM, surface markers by WB	Intrathecal injection, 10 μg, single dose, followed by 7 days until the endpoint	Clinical score: 0–5 scale Pathological score: Inflammation (H&E staining) and demyelination (LFB staining) in the spinal cord Neuroinflammation: Cytokines via ELISA (TNFα, IL-1β, IL-6, IL-10, IL-18) in the spinal cord
Wu J et al. 2025 [75]	EAE, C57BL/6 mice, 6/group, female, 6–8 weeks old	Bone marrow mesenchymal stem cells (BM-MSCs) from C57BL/6 mice	40–160 nm by TEM, surface markers by WB	Intranasal infusion, 100 μg, one dose every other day for 9 days, followed by 14 days until the endpoint	Clinical score: 0–5 scale
Zhang J et al. 2022 [76]	EAE, C57BL/6 mice, 10/group or 6/group, female, 6–8 weeks old	Bone marrow mesenchymal stem cells (BM-MSCs) from rhesus monkey	30–150 nm by TEM and qNano TRPS, surface markers by WB	Intravenous (tail vein) injection, 5 × 10^10^ particles, twice a week for 28 days, followed by the endpoint	Clinical score: 0–5 scale Myelin repair: Expression of myelin proteins via WB (MBP) in the spinal cord Neuroinflammation: Cytokines via WB (TNFα, IL-1β, IL-10) in the spinal cord

Abbreviations: EAE = experimental autoimmune encephalomyelitis; CPZ = Cuprizone; TMEV = Theiler’s murine encephalomyelitis virus; LPC = lysolecithin; H&E = hematoxylin and eosin; LFB = Luxol fast blue; OPCs = oligodendrocyte progenitor cells; OLGs = oligodendrocytes; IHC = immunohistochemistry; WB = Western blotting; ELISA = enzyme-linked immunosorbent assay; AFM = atomic force microscopy; Cryo-EM = cryo-electron microscopy; TEM = transmission electron microscopy; SEM = scanning electron microscopy; DLS = dynamic light scattering; NTA = nanoparticle tracking analysis; LC = liquid chromatography; MSpec = mass spectrometry; Raman spec = Raman spectroscopy; qRT-PCR = quantitative reverse transcription–polymerase chain reaction; RNAseq = RNA sequencing; TRPS = tunable resistive pulse sensing; FACS = fluorescence-activated cell sorting; BCA = bicinchoninic acid; PDGFRα = platelet-derived growth factor receptor alpha; PLP = proteolipid protein; MBP = myelin basic protein; Olig2 = oligodendrocyte transcription factor 2; TNFα = tumor necrosis factor alpha; IFNγ = interferon gamma; Ref. [48] * Equivalent to 0–5 scale, (where 5 = exitus). Markers assessed via qRT-PCR are written in italics. For all studies, clinical scores were assessed daily for the entire duration of the experiment after induction of the MS model, while all other outcomes were assessed post-mortem (after the endpoint).

**Table 2:** Summary of engineered (enhanced) EVs: origins and enhancement-associated strategies aiming to improve therapeutic effects in animal models of MS (data extracted from the selected studies presented in Table 1).

Article	EV Origin	EV Enhancement Strategy	Experimental Effect(s)
Fan J et al. 2023 [62]	Bone marrow mesenchymal stem cells (BM-MSCs) from C57BL/6 mice	Cargo (miRNA): miR-367-p	Alleviates EAE severity
He J et al. 2024 [43]	HEK293 cells	Cargo (protein): Sox10	Promotes OPC differentiation Promotes myelin repair
Hosseini Shamili F et al. 2019 [44]	Bone marrow mesenchymal stem cells (BM-MSCs) from C57BL/6 mice	Cargo (aptamer conjugation): LJM-3064	Alleviates EAE severity
Li Y et al. 2024 * [45]	HEK293 cells	Cargo (protein): Olig2	Promotes myelin repair
Liu SS et al. 2025 [46]	Bone marrow mesenchymal stem cells (BM-MSCs) from C57BL/6 mice	Preconditioning (treatment): Chinese medicinal plant formula-containing serum	Alleviates CPZ demyelination Promotes OPC differentiation
Lombardi M et al. 2019 [67]	Microglia primary cultures from Sprague–Dawley rats	Preconditioning (transwell culture): IL4 exposure	Promotes myelin repair
Park SM et al. 2024 [47]	Adipose tissue mesenchymal stem cells (AdMSCs) from canine samples	Preconditioning (treatment): Hypoxia-mimetic agent deferoxamine	Alleviates EAE severity Modulates neuroinflammation
Qin D et al. 2024 [69]	Umbilical cord mesenchymal stem cells (UC-MSCs) from human samples	Cargo (miRNA): miR-23a-3p	Alleviates EAE severity Promotes myelin repair
Riazifar M et al. 2019 [48]	Bone marrow mesenchymal stem cells (BM-MSCs) from human samples	Preconditioning (treatment): IFNγ exposure	Alleviates EAE severity
Shi Z et al. 2024 [72]	Bone marrow mesenchymal stem cells (BM-MSCs), n/a	Cargo (miRNA): miR-181a-5p	Alleviates EAE severity Modulates neuroinflammation
Wang J et al. 2023 [74]	Bone marrow mesenchymal stem cells (BM-MSCs) from C57BL/6 mice	Cargo (miRNA): miR-23b-3p	Alleviates EAE severity Modulates neuroinflammation

* Not included in data analysis for experimentally enhanced EV conditions due to not fulfilling the minimum number of articles (*n* = 3) per analyzed parameter.

## Data Availability

Supplementary data related to this study are available from the corresponding author upon reasonable request.

## Supplementary Materials

The following supporting information can be downloaded at https://www.mdpi.com/article/10.3390/biom16101454/s1: Figure S1: Overview of the frequency of techniques employed by included papers in studying molecular markers of inflammation (A) and remyelination (B); Table S1: Summary of molecular markers assessed within the meta-analysis and the extent of investigation into EV effect; Figure S2: Funnel plots of clinical scores for studies using native (A) and enhanced (B) EV preparations (all scores were scaled to a 0 to 5 interval of values); Figure S3: Funnel plots of pathological scores of inflammation for studies using native (A) and enhanced (B) EV preparations (all scores were scaled to a 0 to 3 interval of values); Figure S4: Funnel plots of pathological scores of demyelination for studies using native (A) and enhanced (B) EV preparations (all scores were scaled to a 0 to 3 interval of values); Figure S5: Risk of experimental bias in the studies included in the meta-analysis; Figure S6: Selection model analysis of sensitivity to publication bias of meta-analysis outcome clinical score (5-point system) with native EVs performed in JASP v 0.98.1 using *p*-value cut-offs of 0.025 and 0.975 with a fixed effects and random effects inference model: results tables and graph illustrating changes in effect estimate and confidence interval; Figure S7: Selection model analysis of sensitivity to publication bias of meta-analysis outcome clinical score (5-point system) with enhanced EVs performed in JASP v 0.98.1 using *p*-value cut-offs of 0.025 and 0.975 with a fixed effects and random effects inference model: results tables and graph illustrating changes in effect estimate and confidence interval; Figure S8: Selection model analysis of sensitivity to publication bias of meta-analysis outcome pathological score for inflammation with native EVs performed in JASP v 0.98.1 using *p*-value cutoffs of 0.025 and 0.975 with a fixed effects and random effects inference model: results tables and graph illustrating changes in effect estimate and confidence interval; Figure S9: Selection model analysis of sensitivity to publication bias of meta-analysis outcome pathological score for inflammation with enhanced EVs performed in JASP v 0.98.1 using *p*-value cut-offs of 0.025 and 0.975 with a random effects inference model: results table; Figure S10: Selection model analysis of sensitivity to publication bias of meta-analysis outcome pathological score for demyelination with native EVs performed in JASP v 0.98.1 using *p*-value cut-offs of 0.025 and 0.975 with a fixed effects and random effects inference model: results tables and graph illustrating changes in effect estimate and confidence interval; Figure S11: Selection model analysis of sensitivity to publication bias of meta-analysis outcome pathological score for demyelination with enhanced EVs performed in JASP v 0.98.1 using *p*-value cut-offs of 0.025 and 0.975 with a fixed effects and random effects inference model: results tables and graph illustrating changes in effect estimate and confidence interval; Figure S12: Selection model analysis of sensitivity to publication bias of meta-analysis outcome TNFα expression with native EVs performed in JASP v 0.98.1 using *p*-value cut-offs of 0.025 and 0.975 with a fixed effects and random effects inference model: results tables and graph illustrating changes in effect estimate and confidence interval; Figure S13 Selection model analysis of sensitivity to publication bias of meta-analysis outcome TNFα expression with enhanced EVs performed in JASP v 0.98.1 using *p*-value cut-offs of 0.025 and 0.975 with a fixed effects and random effects inference model: results tables and graph illustrating changes in effect estimate and confidence interval; Figure S14: Selection model analysis of sensitivity to publication bias of meta-analysis outcome IFNγ expression with native EVs performed in JASP v 0.98.1 using *p*-value cut-offs of 0.025 and 0.975 with a fixed effects and random effects inference model: results tables and graph illustrating changes in effect estimate and confidence interval; Figure S15: Selection model analysis of sensitivity to publication bias of meta-analysis outcome IL1β expression with native EVs performed in JASP v 0.98.1 using *p*-value cut-offs of 0.025 and 0.975 with a fixed effects and random effects inference model: results tables and graph illustrating changes in effect estimate and confidence interval; Figure S16: Selection model analysis of sensitivity to publication bias of meta-analysis outcome IL1β expression with enhanced EVs performed in JASP v 0.98.1 using *p*-value cut-offs of 0.025 and 0.975 with a fixed effects and random effects inference model: results tables and graph illustrating changes in effect estimate and confidence interval; Figure S17: Selection model analysis of sensitivity to publication bias of meta-analysis outcome IL6 expression with native EVs performed in JASP v 0.98.1 using *p*-value cut-offs of 0.025 and 0.975 with a fixed effects and random effects inference model: results tables and graph illustrating changes in effect estimate and confidence interval; Figure S18: Selection model analysis of sensitivity to publication bias of meta-analysis outcome IL6 expression with enhanced EVs performed in JASP v 0.98.1 using *p*-value cut-offs of 0.025 and 0.975 with a fixed effects and random effects inference model: results tables and graph illustrating changes in effect estimate and confidence interval; Figure S19: Selection model analysis of sensitivity to publication bias of meta-analysis outcome IL10 expression with native EVs performed in JASP v 0.98.1 using *p*-value cut-offs of 0.025 and 0.975 with a fixed effects and random effects inference model: results tables and graph illustrating changes in effect estimate and confidence interval; Figure S20: Selection model analysis of sensitivity to publication bias of meta-analysis outcome IL18 expression with native EVs performed in JASP v 0.98.1 using *p*-value cut-offs of 0.025 and 0.975 with a fixed effects and random effects inference model: results tables and graph illustrating changes in effect estimate and confidence interval; Figure S21: Selection model analysis of sensitivity to publication bias of meta-analysis outcome PDGFRα+ OPCs with native EVs performed in JASP v 0.98.1 using *p*-value cut-offs of 0.025 and 0.975 with a fixed effects and random effects inference model: results tables and graph illustrating changes in effect estimate and confidence interval; Figure S22: Selection model analysis of sensitivity to publication bias of meta-analysis outcome Olig2 with native EVs performed in JASP v 0.98.1 using *p*-value cut-offs of 0.025 and 0.975 with a fixed effects and random effects inference model: results tables and graph illustrating changes in effect estimate and confidence interval; Figure S23: Selection model analysis of sensitivity to publication bias of meta-analysis outcome MBP with native EVs performed in JASP v 0.98.1 using *p*-value cut-offs of 0.025 and 0.975 with a fixed effects and random effects inference model: results tables and graph illustrating changes in effect estimate and confidence interval; Figure S24: Selection model analysis of sensitivity to publication bias of meta-analysis outcome MBP with enhanced EVs performed in JASP v 0.98.1 using *p*-value cut-offs of 0.025 and 0.975 with a fixed effects and random effects inference model: results tables and graph illustrating changes in effect estimate and confidence interval; Figure S25: Selection model analysis of sensitivity to publication bias of meta-analysis outcome PLP expression with native EVs performed in JASP v 0.98.1 using *p*-value cut-offs of 0.025 and 0.975 with a fixed effects and random effects inference model: results tables and graph illustrating changes in effect estimate and confidence interval; Figure S26: Selection model analysis of sensitivity to publication bias of meta-analysis outcome Black-Gold myelin % per field with native EVs performed in JASP v 0.98.1. using *p*-value cut-offs of 0.025 and 0.975 with a fixed effects and random effects inference model: results tables and graph illustrating changes in effect estimate and confidence interval.

## Abbreviations

The following abbreviations are used in this manuscript:

AdMSCs	Adipose tissue mesenchymal stem cells
AKT	Protein kinase B
AFM	Atomic force microscopy
BBB	Blood–brain barrier
BCA	Bicinchoninic acid
BM-MSCs	Bone marrow mesenchymal stem cells
CD	Cluster of differentiation
CI	Confidence interval
CNS	Central nervous system
CPZ	Cuprizone
Cryo-EM	Cryo-electron microscopy
DLS	Dynamic light scattering
DMTs	Disease-modifying therapies
EAE	Experimental autoimmune encephalomyelitis
EBNA1	Epstein–Barr nuclear antigen 1
EBV	Epstein–Barr virus
ELISA	Enzyme-linked immunosorbent assay
EVs	Extracellular vesicles
FACS	Fluorescence-activated cell sorting
H&E	Hematoxylin and eosin
HLA	Human leukocyte antigen
IFNγ	Interferon gamma
IHC	Immunohistochemistry
IL	Interleukin
ISEV	International Society for Extracellular Vesicles
LC	Liquid chromatography (coupled with mass spectrometry)
LFB	Luxol fast blue
LPC	Lysophosphatidylcholine
MBP	Myelin basic protein
MD	Mean difference
miRNA, miR	Micro-ribonucleic acid
MOG	Myelin oligodendrocyte glycoprotein
MS	Multiple sclerosis
MSCs	Mesenchymal stem cell
MSpec	Mass spectrometry
NF-κB	Nuclear factor kappa-light-chain-enhancer of activated B cells
NTA	Nanoparticle tracking analysis
Olig2	Oligodendrocyte transcription factor 2
OPCs	Oligodendrocyte progenitor cells
PDGFRα	Platelet-derived growth factor receptor alpha
PI3K	Phosphoinositide 3-kinase
PLP	Proteolipid protein
PRISMA	Preferred Reporting Items for Systematic Reviews and Meta-Analyses
qRT-PCR	Quantitative real-time reverse transcription–polymerase chain reaction
SEM	Scanning electron microscopy
SMD	Standardized mean difference
Sox10	SRY-related HMG-box gene 10
TEM	Transmission electron microscopy
TLR4	Toll-like receptor 4
TMEV	Theiler’s murine encephalomyelitis virus
TNFα	Tumor necrosis factor alpha
Treg	Regulatory T cell
TRPS	Tunable resistive pulse sensing
UCB-MSCs	Umbilical cord blood plasma mesenchymal stem cells
UC-MSCs	Umbilical cord mesenchymal stem cells
WB	Western blotting
WoS	Web of Science
