# Supplementary material for: Effectiveness of Native and Enhanced Extracellular Vesicle Preparations Assessed in Animal Models of Multiple Sclerosis: A Meta-Analysis
Source: Biomolecules. 2026 Oct 5;16(10):1454. doi: 10.3390/biom16101454 (PMC13702491; doi:10.3390/biom16101454)
Supplement: Supplementary file 1 [file biomolecules-16-01454-s001.zip › biomolecules-4557045-supplementary.pdf]

Supplementary Materials

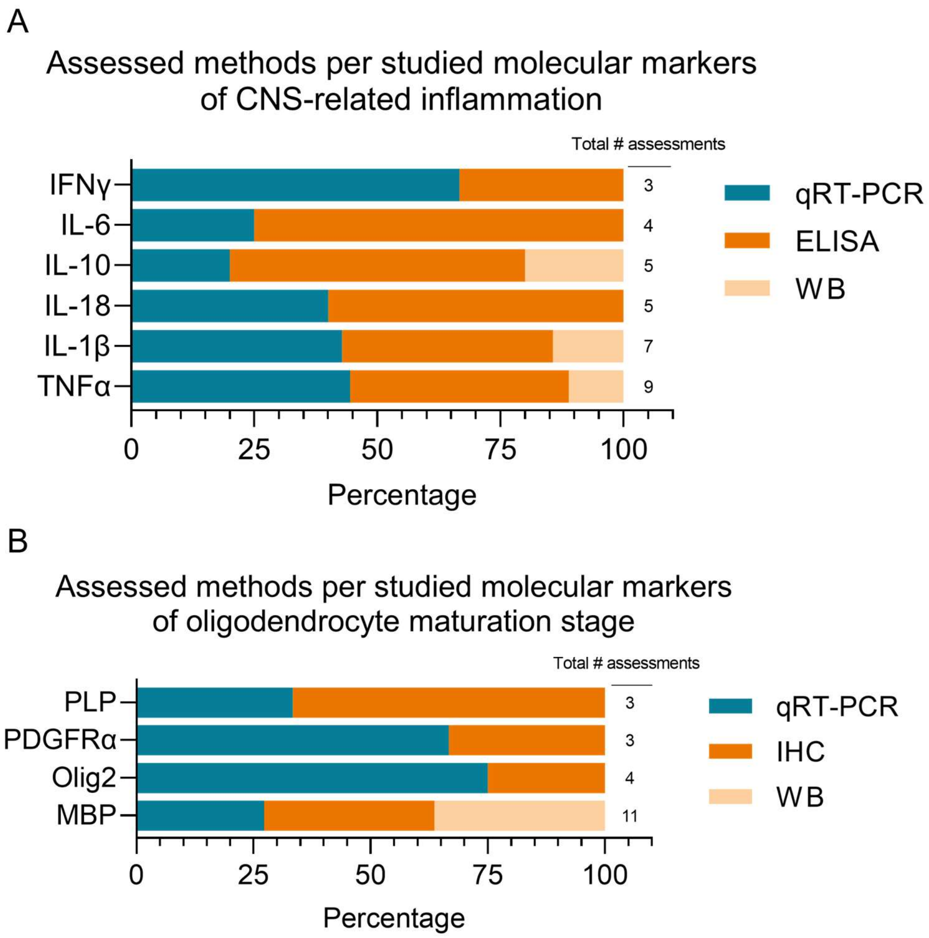

Figure S1. Overview of the frequency of techniques employed by included papers in studying molecular markers of inflammation (A) and remyelination (B). Markers are ordered from least (IFNγ, PLP) to most studied (TNFα, MBP); the data are provided in Table 1, based on the records of the included papers in the meta-analysis. For inflammation markers most of the studies employed antibody-based methods, except for IFNγ quantification (A). For remyelination, the applied techniques vary widely per each marker studied (B). Abbreviations: IFNγ = interferon gamma, IL = interleukin, TNFα = tumour necrosis factor alpha, PDGFRα = platelet-derived growth factor receptor alpha, PLP = proteolipid protein, MBP = myelin basic protein, Olig2 = oligodendrocyte transcription factor 2, IHC = immunohistochemistry, WB = Western blotting, ELISA = enzyme-linked immunosorbent assay, qRT-PCR = quantitative reverse transcription–polymerase chain reaction

Table S1. Summary of molecular markers assessed within the meta-analysis and the extent of investigation into EV effect.

CNS-related inflammation			
Marker	SMD, 95% CI Native EVs	SMD, 95% CI Enhanced EVs	EV effect
TNFα	-3.99 [-5.72, -2.26]	- 8.85 [-11.25, -6.46]	Pro-inflammatory; lower values represent better outcome
IFNγ	-3.07 [-4.81, -1.32]	-	Pro-inflammatory; lower values represent better outcome
IL-1β	-4.08 [-5.84, -2.33]	-5.85 [-7.89, -3.81]	Pro-inflammatory; lower values represent better outcome
IL-6	-6.87 [-11.02, -2.71]	-7.02 [-11.15, -2.89]	Pro-inflammatory; lower values represent better outcome
IL-10	4.27 [2.86, 5.67]	-	Neuroprotective; higher values represent better outcome
IL-18	-3.12 [-4.71, -1.53]	-	Pro-inflammatory; lower values represent better outcome

Oligodendrocyte maturation stage			
Marker	SMD, 95% CI Native EVs	SMD, 95% CI Enhanced EVs	EV effect
PDGFRα	-0.74 [-3.71, 2.24]	-	Immature OPC (1st stage); lower values represent better outcome
Olig2	4.91 [1.81, 8.01]	-	Pre-oligodendrocyte or late OPC (2nd stage) and immature oligodendrocyte (3rd stage); higher values represent better outcome
MBP	2.68 [1.73, 3.63]	5.58 [1.06, 10.09]	Myelinating oligodendrocyte (4th stage); higher values represent better outcome
PLP	7.80 [-1.28, 16.87]	-	Myelinating oligodendrocyte (4th stage); higher values represent better outcome

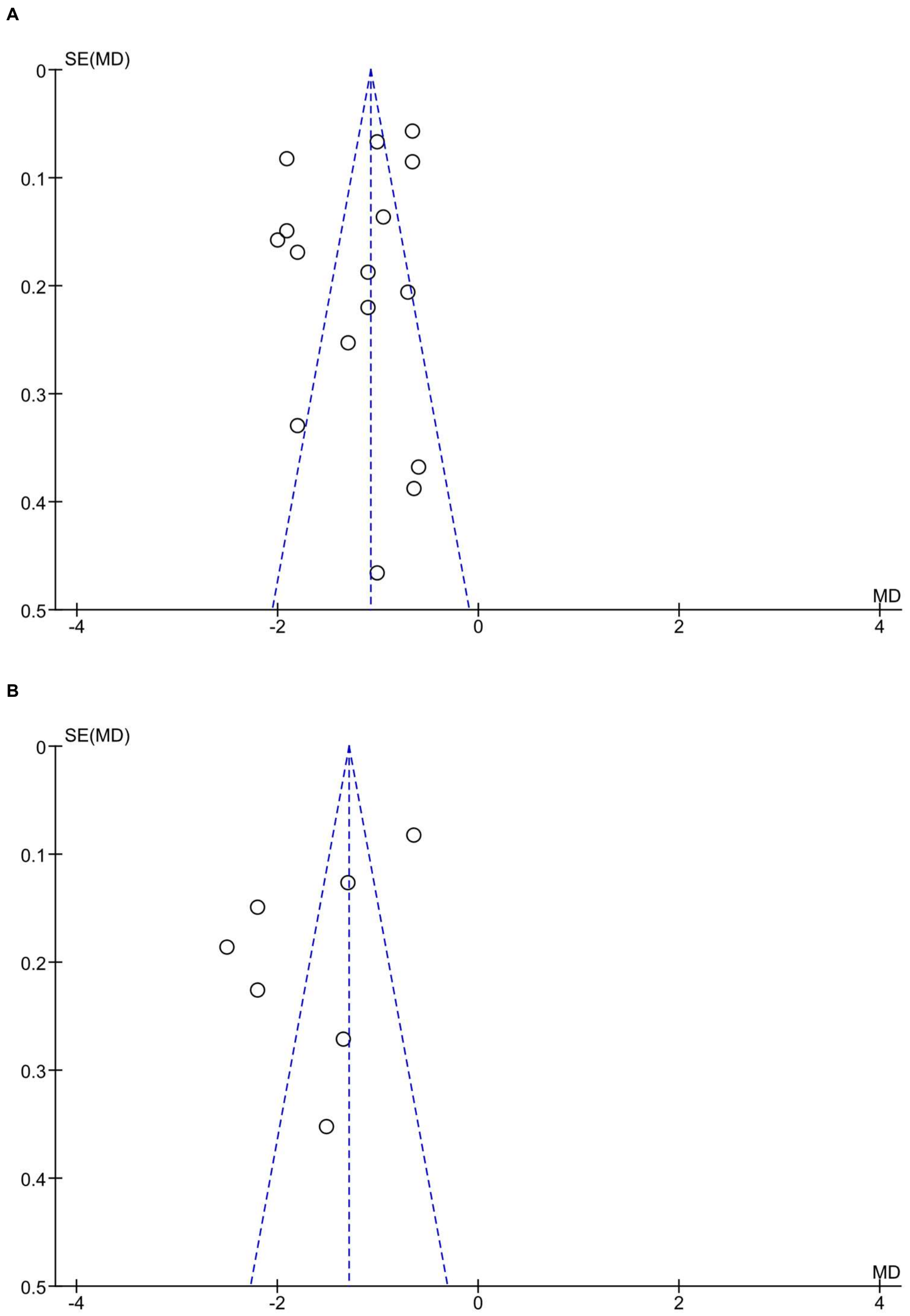

Figure S2. Funnel plots of clinical scores for studies using native (A) and enhanced (B) EV preparations (all scores were scaled to a 0 to 5 interval of values).

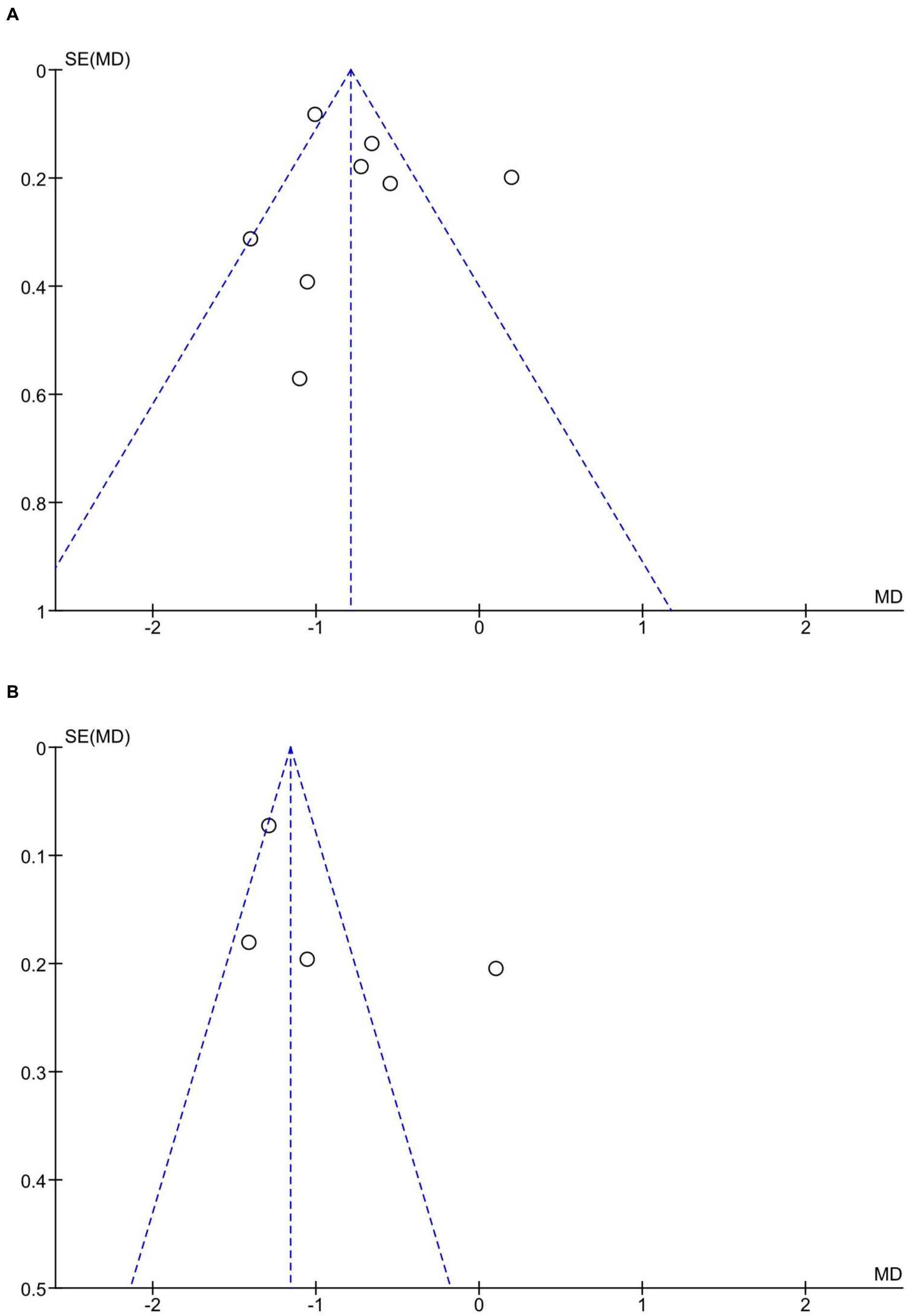

Figure S3. Funnel plots of pathological scores of inflammation for studies using native (A) and enhanced (B) EV preparations (all scores were scaled to a 0 to 3 interval of values).

A

B

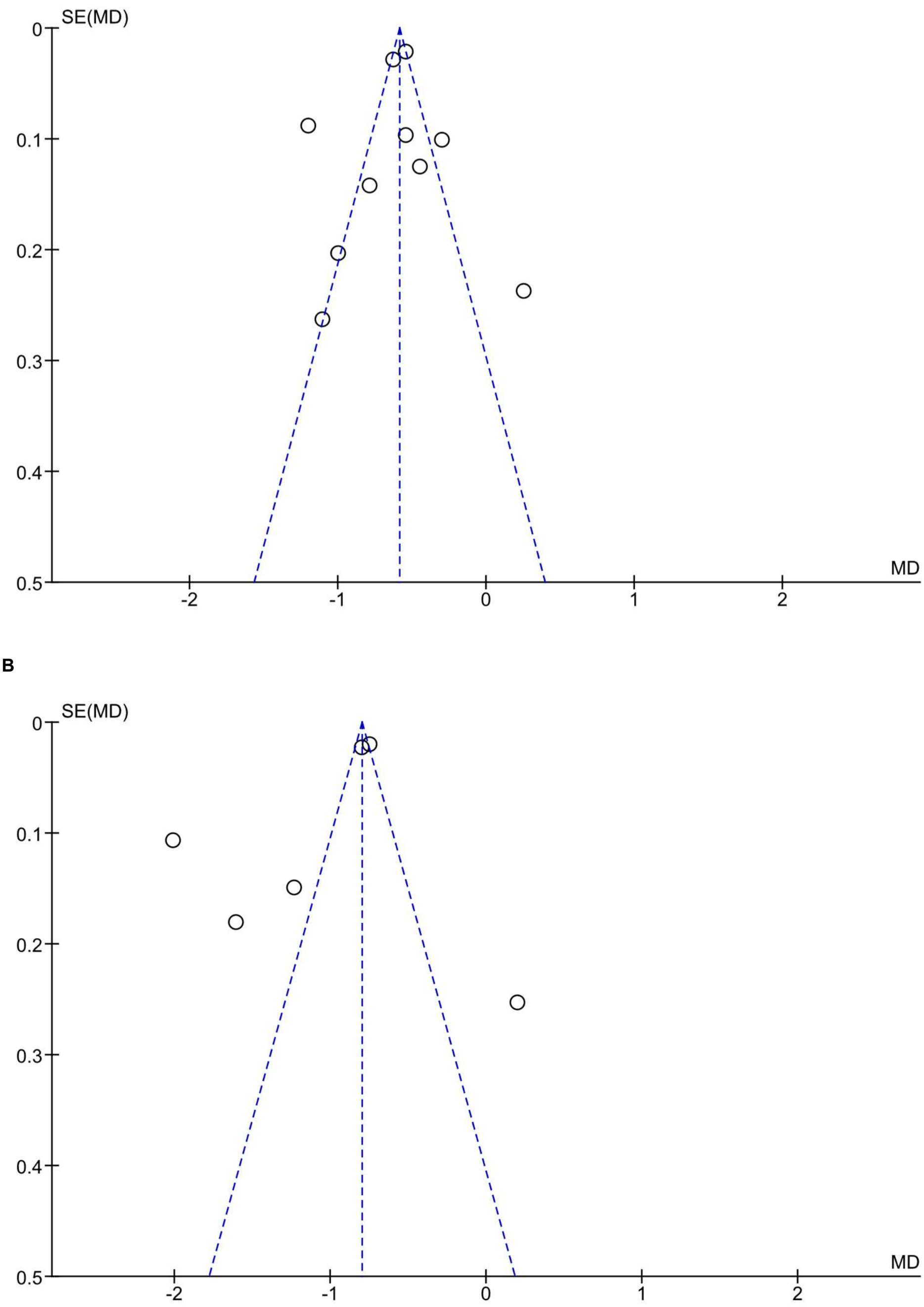

Figure S4. Funnel plots of pathological scores of demyelination for studies using native (A) and enhanced (B) EV preparations (all scores were scaled to a 0 to 3 interval of values).

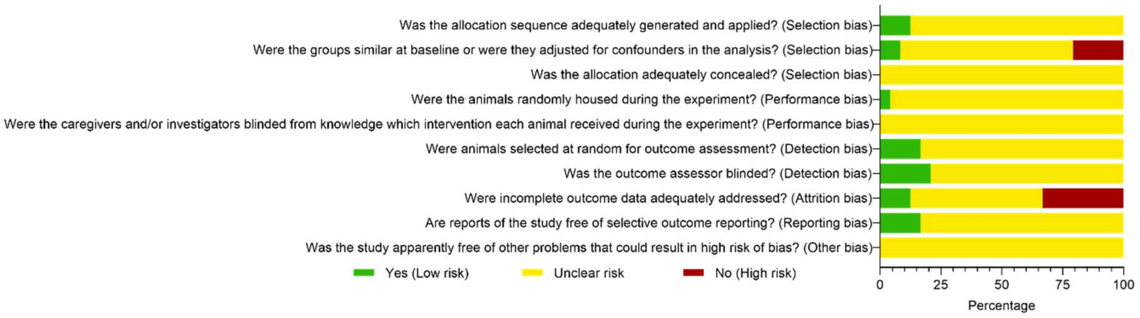

Figure S5. Risk of experimental bias of the studies included in the meta-analysis. Adherence scores for animal research in studies assessing native and enhanced EVs in animal models of multiple sclerosis, via SYRCLE items, in agreement with the Cochrane Risk of Bias tool.

Fixed Effects Estimates

Mean Estimates (μ)

	Estimate	Standard Error	z	p	95% Confidence Interval	
					Lower	Upper
Unadjusted	−1.077	0.03011	−35.77	< .001	−1.136	−1.018
Adjusted	−1.085	0.02870	−37.80	< .001	−1.141	−1.029

Warning: At least one of the p-value intervals contains no effect sizes, leading to estimation problems. Consider re-specifying the cutoffs.

Random Effects Estimates

Mean Estimates (μ)

	Estimate	Standard Error	z	p	95% Confidence Interval	
					Lower	Upper
Unadjusted	−1.211	0.1300	−9.317	< .001	−1.466	−0.9564
Adjusted	−1.301	0.09876	−13.17	< .001	−1.494	−1.107

Warning: At least one of the p-value intervals contains no effect sizes, leading to estimation problems. Consider re-specifying the cutoffs.

Mean Model Estimates (μ)

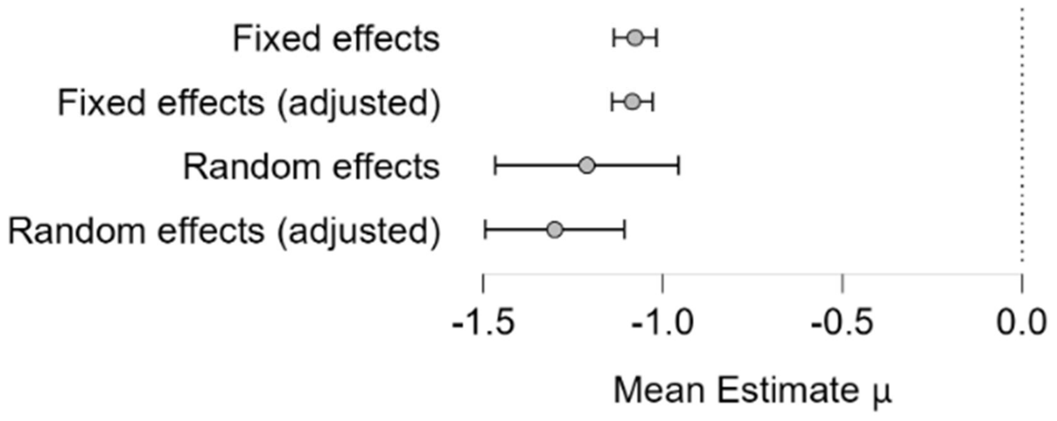

Figure S6. Selection model analysis of sensitivity to publication bias of meta-analysis outcome **clinical score (5-point system) with native EVs** performed in JASP v 0.98.1 using P-value cut-offs of 0.025 and 0.975 with a fixed effects and random effects inference model: results tables and graph illustrating changes in effect estimate and confidence interval.

Fixed Effects Estimates

Mean Estimates (μ)

	Estimate	Standard Error	z	p	95% Confidence Interval	
					Lower	Upper
Unadjusted	−1.290	0.05556	−23.22	< .001	−1.399	−1.181
Adjusted	−1.288	0.05174	−24.90	< .001	−1.390	−1.187

Warning: At least one of the p-value intervals contains no effect sizes, leading to estimation problems. Consider re-specifying the cutoffs.

Random Effects Estimates

Mean Estimates (μ)

	Estimate	Standard Error	z	p	95% Confidence Interval	
					Lower	Upper
Unadjusted	−1.663	0.2416	−6.883	< .001	−2.136	−1.189
Adjusted	−1.577	0.1631	−9.670	< .001	−1.896	−1.257

Warning: At least one of the p-value intervals contains no effect sizes, leading to estimation problems. Consider re-specifying the cutoffs.

Mean Model Estimates (μ)

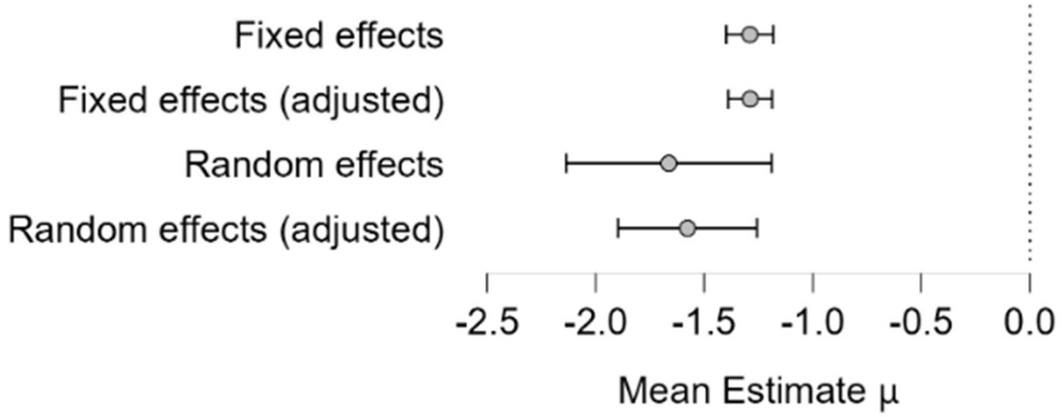

Figure S7. Selection model analysis of sensitivity to publication bias of meta-analysis outcome **clinical score (5-point system) with enhanced EVs** performed in JASP v 0.98.1 using P-value cut-offs of 0.025 and 0.975 with a fixed effects and random effects inference model: results tables and graph illustrating changes in effect estimate and confidence interval.

Fixed Effects Estimates

Mean Estimates (μ)

	Estimate	Standard Error	z	p	95% Confidence Interval	
					Lower	Upper
Unadjusted	−0.7898	0.05800	−13.62	< .001	−0.9035	−0.6761
Adjusted	−0.8019	0.05083	−15.78	< .001	−0.9015	−0.7023

Note: 48 observations were removed due to missing values.

Warning: At least one of the p-value intervals contains no effect sizes, leading to estimation problems. Consider re-specifying the cutoffs.

Random Effects Estimates

Mean Estimates (μ)

	Estimate	Standard Error	z	p	95% Confidence Interval	
					Lower	Upper
Unadjusted	−0.7270	0.1626	−4.471	< .001	−1.046	−0.4083
Adjusted	−0.6450	0.1138	−5.666	< .001	−0.8681	−0.4219

Note: 48 observations were removed due to missing values.

Warning: At least one of the p-value intervals contains no effect sizes, leading to estimation problems. Consider re-specifying the cutoffs.

Mean Model Estimates (μ)

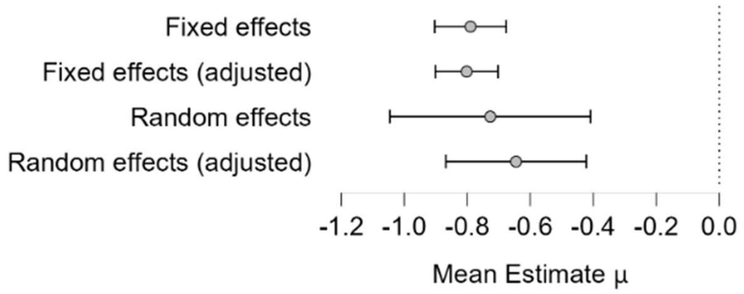

Figure S8. Selection model analysis of sensitivity to publication bias of meta-analysis outcome **pathological score for inflammation with native EVs** performed in JASP v 0.98.1 using P-value cut-offs of 0.025 and 0.975 with a fixed effects and random effects inference model: results tables and graph illustrating changes in effect estimate and confidence interval.

Random Effects Estimates

Mean Estimates (μ)

	Estimate	Standard Error	z	p	95% Confidence Interval	
					Lower	Upper
Unadjusted	−0.9264	0.2933	−3.159	.002	−1.501	−0.3516
Adjusted	−1.136	0.2407	−4.722	< .001	−1.608	−0.6646

Warning: At least one of the p-value intervals contains no effect sizes, leading to estimation problems. Consider re-specifying the cutoffs.

Figure S9. Selection model analysis of sensitivity to publication bias of meta-analysis outcome **pathological score for inflammation with enhanced EVs** performed in JASP v 0.98.1 using P-value cut-offs of 0.025 and 0.975 with a random effects inference model: results table.

Fixed Effects Estimates

Mean Estimates (μ)

	Estimate	Standard Error	z	p	95% Confidence Interval	
					Lower	Upper
Unadjusted	−0.5861	0.01602	−36.58	< .001	−0.6175	−0.5547
Adjusted	−0.5875	0.01480	−39.71	< .001	−0.6165	−0.5585

Warning: At least one of the p-value intervals contains no effect sizes, leading to estimation problems. Consider re-specifying the cutoffs.

Random Effects Estimates

Mean Estimates (μ)

	Estimate	Standard Error	z	p	95% Confidence Interval	
					Lower	Upper
Unadjusted	−0.6293	0.1135	−5.547	< .001	−0.8517	−0.4069
Adjusted	−0.5835	0.06493	−8.987	< .001	−0.7108	−0.4563

Warning: At least one of the p-value intervals contains no effect sizes, leading to estimation problems. Consider re-specifying the cutoffs.

Mean Model Estimates (μ)

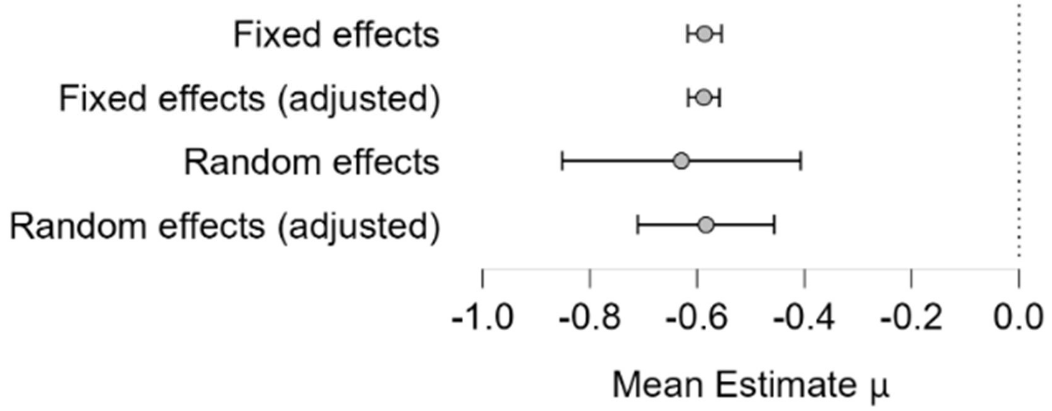

Figure S10. Selection model analysis of sensitivity to publication bias of meta-analysis outcome **pathological score for demyelination with native EVs** performed in JASP v 0.98.1 using P-value cut-offs of 0.025 and 0.975 with a fixed effects and random effects inference model: results tables and graph illustrating changes in effect estimate and confidence interval.

Fixed Effects Estimates

Mean Estimates (μ)

	Estimate	Standard Error	z	p	95% Confidence Interval	
					Lower	Upper
Unadjusted	−0.7980	0.01503	−53.11	< .001	−0.8274	−0.7685
Adjusted	−0.8012	0.01493	−53.67	< .001	−0.8304	−0.7719

Warning: At least one of the p-value intervals contains no effect sizes, leading to estimation problems. Consider re-specifying the cutoffs.

Random Effects Estimates

Mean Estimates (μ)

	Estimate	Standard Error	z	p	95% Confidence Interval	
					Lower	Upper
Unadjusted	−1.043	0.2759	−3.781	< .001	−1.584	−0.5025
Adjusted	−1.246	0.1612	−7.730	< .001	−1.562	−0.9301

Warning: At least one of the p-value intervals contains no effect sizes, leading to estimation problems. Consider re-specifying the cutoffs.

Mean Model Estimates (μ)

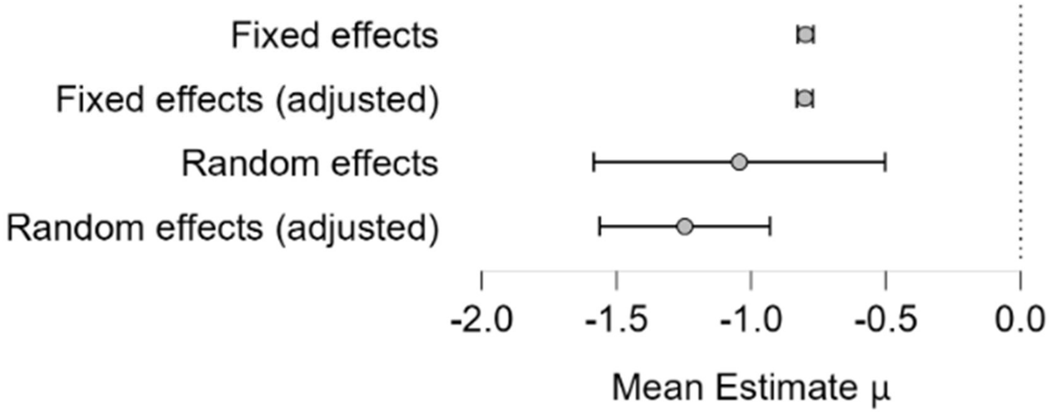

Figure S11. Selection model analysis of sensitivity to publication bias of meta-analysis outcome **pathological score for demyelination with enhanced EVs** performed in JASP v 0.98.1 using P-value cut-offs of 0.025 and 0.975 with a fixed effects and random effects inference model: results tables and graph illustrating changes in effect estimate and confidence interval.

Fixed Effects Estimates

Mean Estimates (μ)

	Estimate	Standard Error	z	p	95% Confidence Interval Lower	Upper
Unadjusted	−2.515	0.2915	−8.626	< .001	−3.086	−1.943
Adjusted	−2.169	0.1184	−18.32	< .001	−2.401	−1.937

Warning: At least one of the p-value intervals contains no effect sizes, leading to estimation problems. Consider re-specifying the cutoffs.

Random Effects Estimates

Mean Estimates (μ)

	Estimate	Standard Error	z	p	95% Confidence Interval Lower	Upper
Unadjusted	−3.836	0.8566	−4.477	< .001	−5.515	−2.157
Adjusted	−2.196	0.3721	−5.902	< .001	−2.926	−1.467

Warning: At least one of the p-value intervals contains no effect sizes, leading to estimation problems. Consider re-specifying the cutoffs.

Mean Model Estimates (μ)

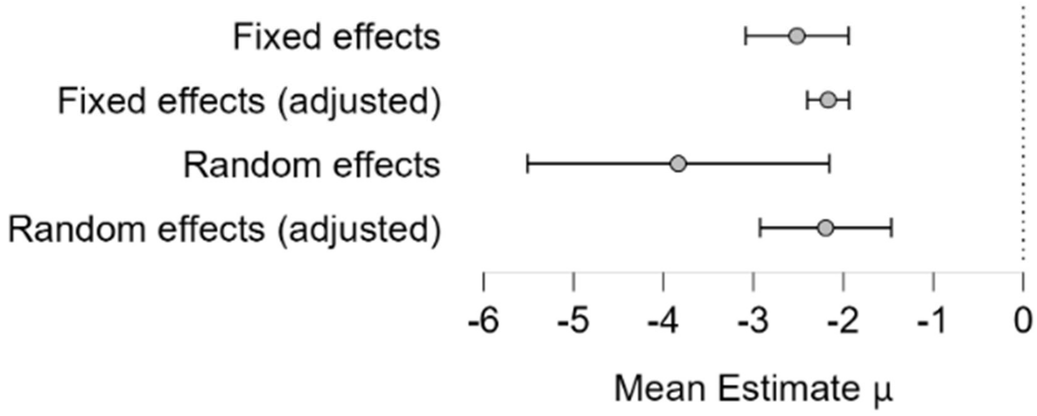

Figure S12. Selection model analysis of sensitivity to publication bias of meta-analysis outcome **TNFα expression with native EVs** performed in JASP v 0.98.1 using P-value cut-offs of 0.025 and 0.975 with a fixed effects and random effects inference model: results tables and graph illustrating changes in effect estimate and confidence interval.

Fixed Effects Estimates

Mean Estimates (μ)

	Estimate	Standard Error	z	p	95% Confidence Interval	
					Lower	Upper
Unadjusted	−8.749	1.118	−7.827	< .001	−10.94	−6.558
Adjusted	−8.649	0.7680	−11.26	< .001	−10.15	−7.144

Warning: At least one of the p-value intervals contains no effect sizes, leading to estimation problems. Consider re-specifying the cutoffs.

Random Effects Estimates

Mean Estimates (μ)

	Estimate	Standard Error	z	p	95% Confidence Interval	
					Lower	Upper
Unadjusted	−8.749	1.353	−6.467	< .001	−11.40	−6.098
Adjusted	−8.649	1.229	−7.037	< .001	−11.06	−6.240

Warning: At least one of the p-value intervals contains no effect sizes, leading to estimation problems. Consider re-specifying the cutoffs.

Mean Model Estimates (μ)

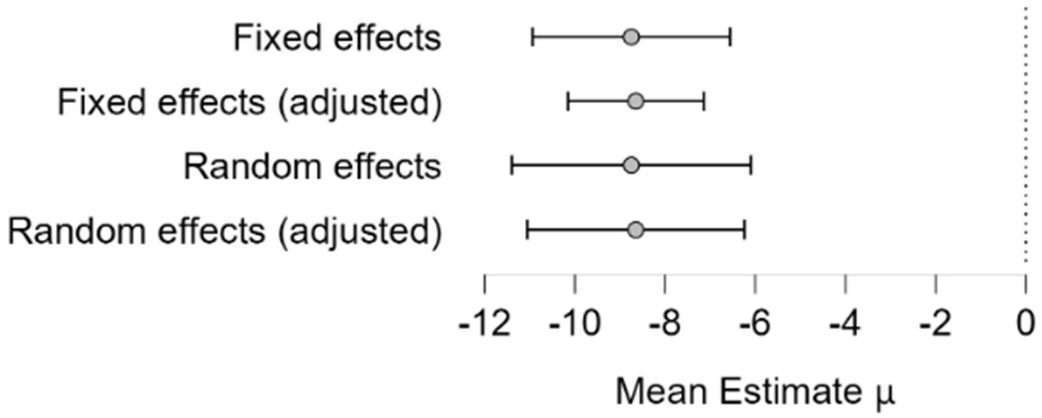

Figure S13 Selection model analysis of sensitivity to publication bias of meta-analysis outcome **TNFα expression with enhanced EVs** performed in JASP v 0.98.1 using P-value cut-offs of 0.025 and 0.975 with a fixed effects and random effects inference model: results tables and graph illustrating changes in effect estimate and confidence interval.

Fixed Effects Estimates

Mean Estimates (μ)

					95% Confidence Interval	
	Estimate	Standard Error	z	p	Lower	Upper
Unadjusted	−2.572	0.4025	−6.389	< .001	−3.361	−1.783
Adjusted	−2.435	0.2064	−11.80	< .001	−2.840	−2.031

Warning: At least one of the p-value intervals contains no effect sizes, leading to estimation problems. Consider re-specifying the cutoffs.

Random Effects Estimates

Mean Estimates (μ)

					95% Confidence Interval	
	Estimate	Standard Error	z	p	Lower	Upper
Unadjusted	−2.978	0.7394	−4.028	< .001	−4.427	−1.529
Adjusted	−2.277	0.5648	−4.030	< .001	−3.384	−1.169

Warning: At least one of the p-value intervals contains no effect sizes, leading to estimation problems. Consider re-specifying the cutoffs.

Mean Model Estimates (μ)

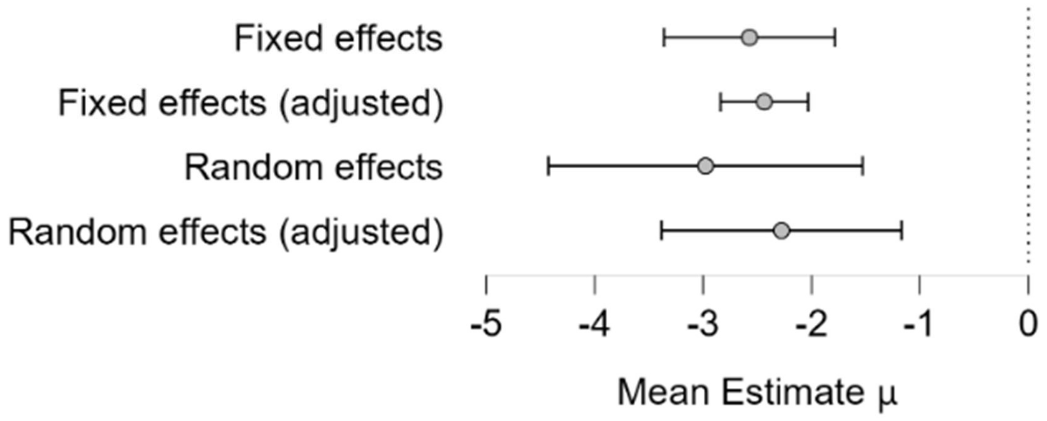

Figure S14. Selection model analysis of sensitivity to publication bias of meta-analysis outcome **IFNγ expression with native EVs** performed in JASP v 0.98.1 using P-value cut-offs of 0.025 and 0.975 with a fixed effects and random effects inference model: results tables and graph illustrating changes in effect estimate and confidence interval.

Fixed Effects Estimates

Mean Estimates (μ)

	Estimate	Standard Error	z	p	95% Confidence Interval	
					Lower	Upper
Unadjusted	−2.881	0.3529	−8.164	< .001	−3.573	−2.189
Adjusted	−2.530	0.1074	−23.55	< .001	−2.741	−2.319

Warning: At least one of the p-value intervals contains no effect sizes, leading to estimation problems. Consider re-specifying the cutoffs.

Random Effects Estimates

Mean Estimates (μ)

	Estimate	Standard Error	z	p	95% Confidence Interval	
					Lower	Upper
Unadjusted	−4.100	0.9361	−4.380	< .001	−5.935	−2.265
Adjusted	−1.313	0.2935	−4.474	< .001	−1.888	−0.7378

Warning: At least one of the p-value intervals contains no effect sizes, leading to estimation problems. Consider re-specifying the cutoffs.

Mean Model Estimates (μ)

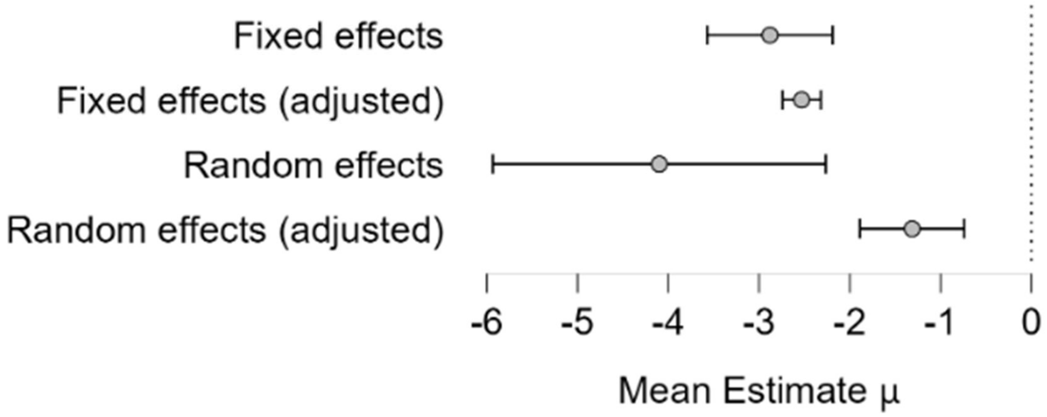

Figure S15. Selection model analysis of sensitivity to publication bias of meta-analysis outcome **IL1β expression with native EVs** performed in JASP v 0.98.1 using P-value cut-offs of 0.025 and 0.975 with a fixed effects and random effects inference model: results tables and graph illustrating changes in effect estimate and confidence interval.

Fixed Effects Estimates

Mean Estimates (μ)

					95% Confidence Interval	
	Estimate	Standard Error	z	p	Lower	Upper
Unadjusted	−5.580	0.7560	−7.381	< .001	−7.061	−4.098
Adjusted	−5.416	0.2062	−26.26	< .001	−5.820	−5.011

Warning: At least one of the p-value intervals contains no effect sizes, leading to estimation problems. Consider re-specifying the cutoffs.

Random Effects Estimates

Mean Estimates (μ)

					95% Confidence Interval	
	Estimate	Standard Error	z	p	Lower	Upper
Unadjusted	−5.647	0.9376	−6.023	< .001	−7.485	−3.810
Adjusted	−5.481	0.3347	−16.38	< .001	−6.137	−4.825

Warning: At least one of the p-value intervals contains no effect sizes, leading to estimation problems. Consider re-specifying the cutoffs.

Mean Model Estimates (μ)

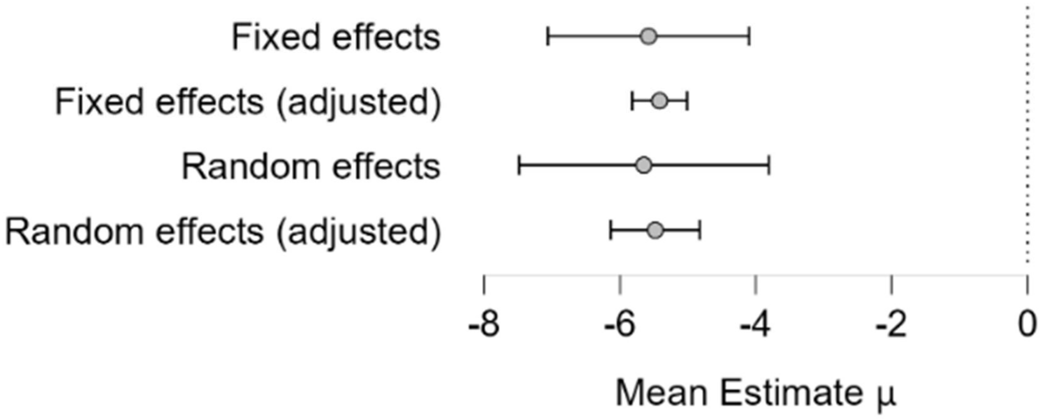

Figure S16. Selection model analysis of sensitivity to publication bias of meta-analysis outcome **IL1β expression with enhanced EVs** performed in JASP v 0.98.1 using P-value cut-offs of 0.025 and 0.975 with a fixed effects and random effects inference model: results tables and graph illustrating changes in effect estimate and confidence interval.

Fixed Effects Estimates

Mean Estimates (μ)

					95% Confidence Interval	
	Estimate	Standard Error	z	p	Lower	Upper
Unadjusted	−2.699	0.4599	−5.868	< .001	−3.600	−1.797
Adjusted	−2.368	0.002014	−1176	< .001	−2.372	−2.364

Warning: At least one of the p-value intervals contains no effect sizes, leading to estimation problems. Consider re-specifying the cutoffs.

Random Effects Estimates

Mean Estimates (μ)

					95% Confidence Interval	
	Estimate	Standard Error	z	p	Lower	Upper
Unadjusted	−10.47	5.765	−1.815	.069	−21.77	0.8332
Adjusted	12.43	0.7943	15.65	< .001	10.87	13.99

Warning: At least one of the p-value intervals contains no effect sizes, leading to estimation problems. Consider re-specifying the cutoffs.

Mean Model Estimates (μ)

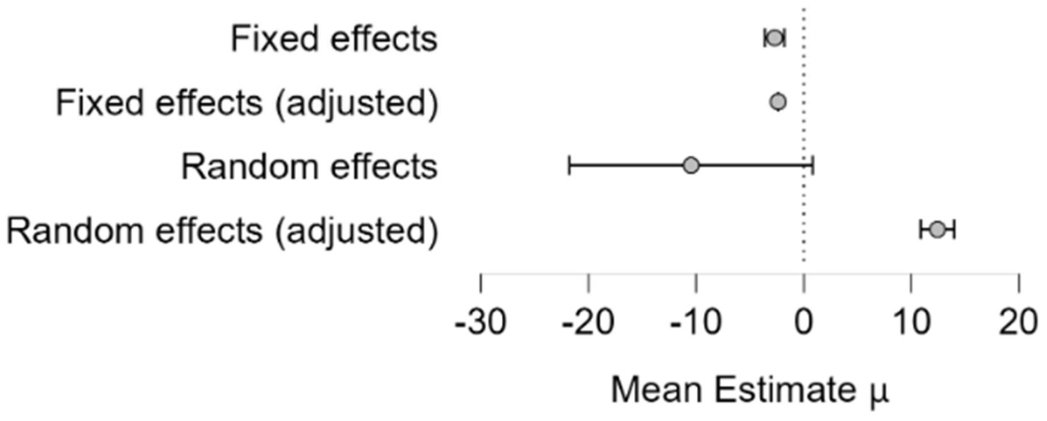

Figure S17. Selection model analysis of sensitivity to publication bias of meta-analysis outcome **IL6 expression with native EVs** performed in JASP v 0.98.1 using P-value cut-offs of 0.025 and 0.975 with a fixed effects and random effects inference model: results tables and graph illustrating changes in effect estimate and confidence interval.

Fixed Effects Estimates

Mean Estimates (μ)

					95% Confidence Interval	
	Estimate	Standard Error	z	p	Lower	Upper
Unadjusted	−5.107	0.7466	−6.841	< .001	−6.571	−3.644
Adjusted	−4.887	0.02146	−227.7	< .001	−4.929	−4.845

Warning: At least one of the p-value intervals contains no effect sizes, leading to estimation problems. Consider re-specifying the cutoffs.

Random Effects Estimates

Mean Estimates (μ)

					95% Confidence Interval	
	Estimate	Standard Error	z	p	Lower	Upper
Unadjusted	−7.139	2.507	−2.847	.004	−12.05	−2.225
Adjusted	−2.097	1.639	−1.279	.201	−5.309	1.116

Warning: At least one of the p-value intervals contains no effect sizes, leading to estimation problems. Consider re-specifying the cutoffs.

Mean Model Estimates (μ)

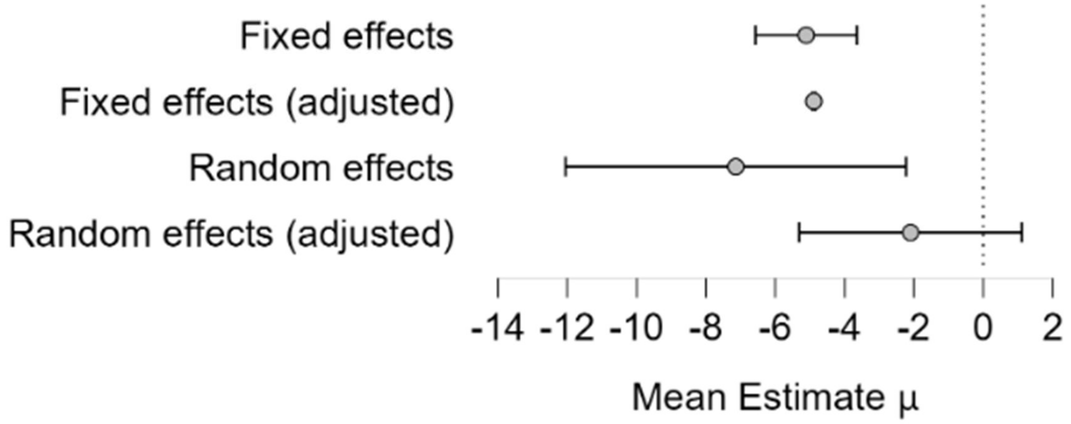

Figure S18. Selection model analysis of sensitivity to publication bias of meta-analysis outcome **IL6 expression with enhanced EVs** performed in JASP v 0.98.1 using P-value cut-offs of 0.025 and 0.975 with a fixed effects and random effects inference model: results tables and graph illustrating changes in effect estimate and confidence interval.

Fixed Effects Estimates

Mean Estimates (μ)

	Estimate	Standard Error	z	p	95% Confidence Interval Lower	95% Confidence Interval Upper
Unadjusted	3.888	0.4437	8.763	< .001	3.018	4.758
Adjusted	3.733	0.1400	26.67	< .001	3.459	4.007

Warning: At least one of the p-value intervals contains no effect sizes, leading to estimation problems. Consider re-specifying the cutoffs.

Random Effects Estimates

Mean Estimates (μ)

	Estimate	Standard Error	z	p	95% Confidence Interval Lower	95% Confidence Interval Upper
Unadjusted	3.888	0.8631	4.505	< .001	2.196	5.580
Adjusted	3.733	0.5085	7.342	< .001	2.737	4.730

Warning: At least one of the p-value intervals contains no effect sizes, leading to estimation problems. Consider re-specifying the cutoffs.

Mean Model Estimates (μ)

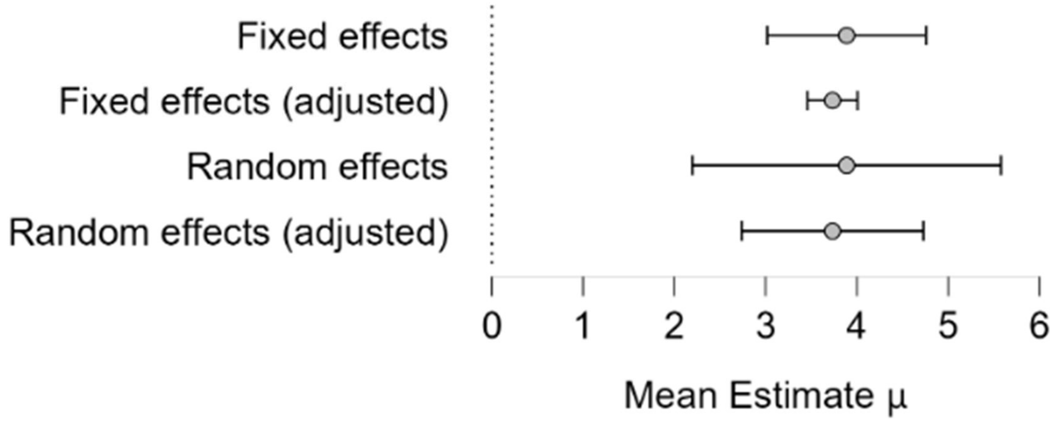

Figure S19. Selection model analysis of sensitivity to publication bias of meta-analysis outcome **IL10 expression with native EVs** performed in JASP v 0.98.1 using P-value cut-offs of 0.025 and 0.975 with a fixed effects and random effects inference model: results tables and graph illustrating changes in effect estimate and confidence interval.

Fixed Effects Estimates

Mean Estimates (μ)

					95% Confidence Interval	
	Estimate	Standard Error	z	p	Lower	Upper
Unadjusted	−2.568	0.3736	−6.875	< .001	−3.301	−1.836
Adjusted	−2.239	0.1749	−12.80	< .001	−2.582	−1.896

Warning: At least one of the p-value intervals contains no effect sizes, leading to estimation problems. Consider re-specifying the cutoffs.

Random Effects Estimates

Mean Estimates (μ)

					95% Confidence Interval	
	Estimate	Standard Error	z	p	Lower	Upper
Unadjusted	−3.158	0.8955	−3.527	< .001	−4.913	−1.403
Adjusted	1.094	0.4343	2.519	.012	0.2428	1.945

Warning: At least one of the p-value intervals contains no effect sizes, leading to estimation problems. Consider re-specifying the cutoffs.

Mean Model Estimates (μ)

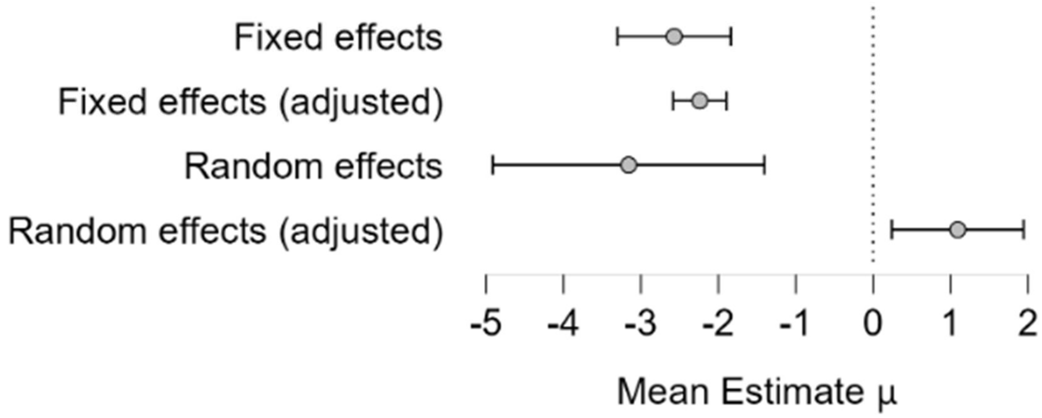

Figure S20. Selection model analysis of sensitivity to publication bias of meta-analysis outcome **IL18 expression with native EVs** performed in JASP v 0.98.1 using P-value cut-offs of 0.025 and 0.975 with a fixed effects and random effects inference model: results tables and graph illustrating changes in effect estimate and confidence interval.

Fixed Effects Estimates

Mean Estimates (μ)

					95% Confidence Interval	
	Estimate	Standard Error	z	p	Lower	Upper
Unadjusted	−0.3663	0.5171	−0.7085	.479	−1.380	0.6471
Adjusted	−0.4774	0.6085	−0.7845	.433	−1.670	0.7153

Warning: At least one of the p-value intervals contains three or fewer effect sizes, which may lead to estimation problems. Consider re-specifying the cutoffs.

Random Effects Estimates ▼

Mean Estimates (μ) ▼

					95% Confidence Interval	
	Estimate	Standard Error	z	p	Lower	Upper
Unadjusted	−0.6307	1.267	−0.4978	.619	−3.114	1.853
Adjusted	−1.023	1.677	−0.6097	.542	−4.310	2.265

Warning: At least one of the p-value intervals contains three or fewer effect sizes, which may lead to estimation problems. Consider re-specifying the cutoffs.

Mean Model Estimates (μ)

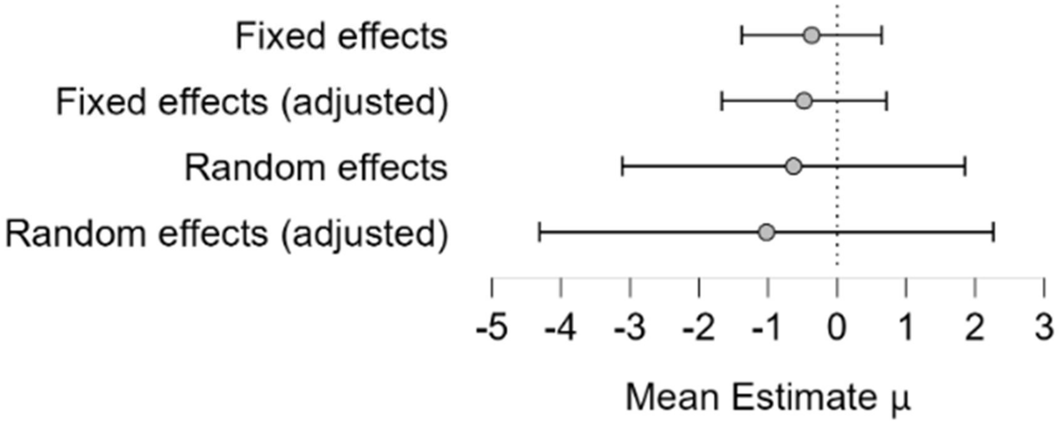

Figure S21. Selection model analysis of sensitivity to publication bias of meta-analysis outcome **PDGFRα+ OPCs with native EVs** performed in JASP v 0.98.1 using P-value cut-offs of 0.025 and 0.975 with a fixed effects and random effects inference model: results tables and graph illustrating changes in effect estimate and confidence interval.

Fixed Effects Estimates

Mean Estimates (μ)

	Estimate	Standard Error	z	p	95% Confidence Interval Lower	Upper
Unadjusted	3.648	0.5606	6.507	< .001	2.549	4.747
Adjusted	3.150	0.001968	1600	< .001	3.146	3.153

Warning: At least one of the p-value intervals contains no effect sizes, leading to estimation problems. Consider re-specifying the cutoffs.

Random Effects Estimates

Mean Estimates (μ)

	Estimate	Standard Error	z	p	95% Confidence Interval Lower	Upper
Unadjusted	5.064	1.957	2.587	.010	1.227	8.900
Adjusted	−3.418	0.1857	−18.40	< .001	−3.781	−3.054

Warning: At least one of the p-value intervals contains no effect sizes, leading to estimation problems. Consider re-specifying the cutoffs.

Mean Model Estimates (μ)

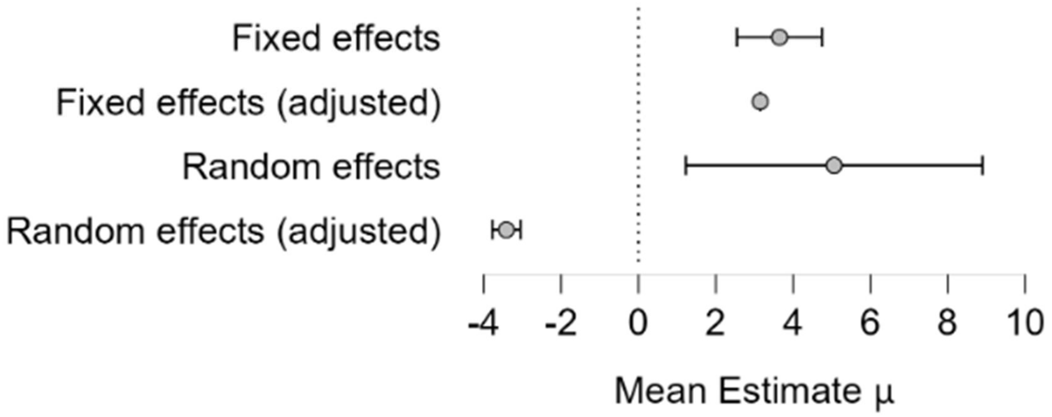

Figure S22. Selection model analysis of sensitivity to publication bias of meta-analysis outcome **Olig2 with native EVs** performed in JASP v 0.98.1 using P-value cut-offs of 0.025 and 0.975 with a fixed effects and random effects inference model: results tables and graph illustrating changes in effect estimate and confidence interval.

Fixed Effects Estimates

Mean Estimates (μ)

					95% Confidence Interval	
	Estimate	Standard Error	z	p	Lower	Upper
Unadjusted	2.182	0.2458	8.877	< .001	1.700	2.663
Adjusted	1.839	0.1649	11.15	< .001	1.516	2.162

Warning: At least one of the p-value intervals contains no effect sizes, leading to estimation problems. Consider re-specifying the cutoffs.

Random Effects Estimates ▼

Mean Estimates (μ) ▼

					95% Confidence Interval	
	Estimate	Standard Error	z	p	Lower	Upper
Unadjusted	2.658	0.4844	5.488	< .001	1.709	3.608
Adjusted	1.394	0.1945	7.168	< .001	1.013	1.775

Warning: At least one of the p-value intervals contains no effect sizes, leading to estimation problems. Consider re-specifying the cutoffs.

Mean Model Estimates (μ)

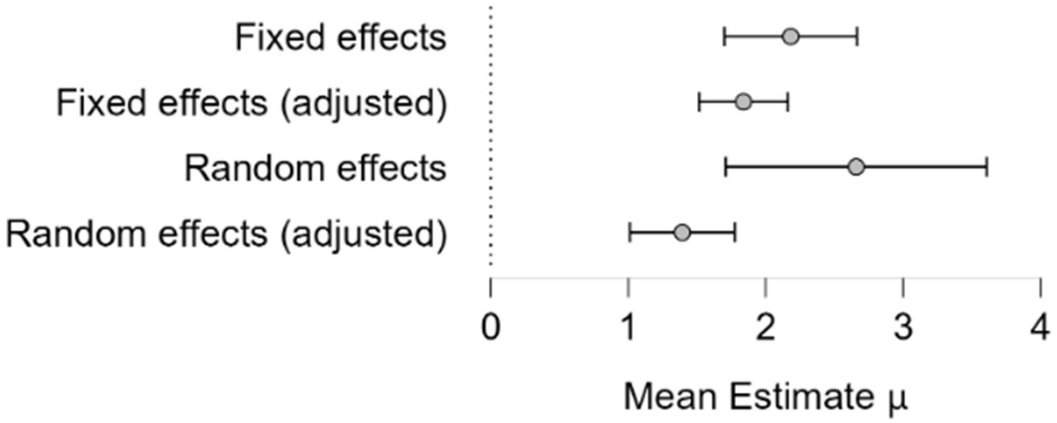

Figure S23. Selection model analysis of sensitivity to publication bias of meta-analysis outcome **MBP with native EVs** performed in JASP v 0.98.1 using P-value cut-offs of 0.025 and 0.975 with a fixed effects and random effects inference model: results tables and graph illustrating changes in effect estimate and confidence interval.

Fixed Effects Estimates

Mean Estimates (μ)

					95% Confidence Interval	
	Estimate	Standard Error	z	p	Lower	Upper
Unadjusted	3.932	0.8099	4.855	< .001	2.345	5.520
Adjusted	3.766	0.05231	72.00	< .001	3.664	3.869

Warning: At least one of the p-value intervals contains no effect sizes, leading to estimation problems. Consider re-specifying the cutoffs.

Random Effects Estimates ▼

Mean Estimates (μ) ▼

					95% Confidence Interval	
	Estimate	Standard Error	z	p	Lower	Upper
Unadjusted	5.152	1.798	2.865	.004	1.627	8.676
Adjusted	4.766	0.9623	4.953	< .001	2.880	6.653

Warning: At least one of the p-value intervals contains no effect sizes, leading to estimation problems. Consider re-specifying the cutoffs.

Mean Model Estimates (μ)

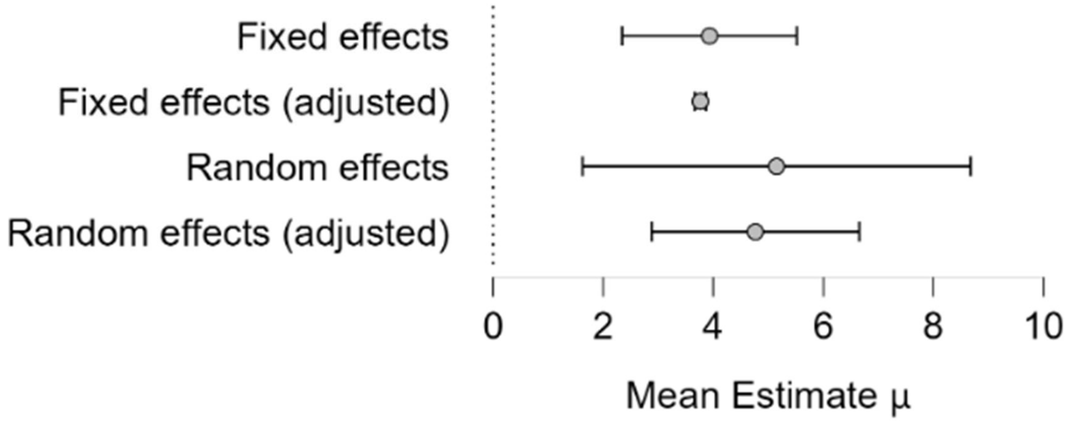

Figure S24. Selection model analysis of sensitivity to publication bias of meta-analysis outcome **MBP with enhanced EVs** performed in JASP v 0.98.1 using P-value cut-offs of 0.025 and 0.975 with a fixed effects and random effects inference model: results tables and graph illustrating changes in effect estimate and confidence interval.

Fixed Effects Estimates

Mean Estimates (μ)

					95% Confidence Interval	
	Estimate	Standard Error	z	p	Lower	Upper
Unadjusted	4.464	1.006	4.438	< .001	2.492	6.435
Adjusted	4.933	0.5756	8.571	< .001	3.805	6.061

Warning: At least one of the p-value intervals contains no effect sizes, leading to estimation problems. Consider re-specifying the cutoffs.

Random Effects Estimates

Mean Estimates (μ)

					95% Confidence Interval	
	Estimate	Standard Error	z	p	Lower	Upper
Unadjusted	7.654	3.359	2.279	.023	1.070	14.24
Adjusted	13.81	3.180	4.342	< .001	7.576	20.04

Warning: At least one of the p-value intervals contains no effect sizes, leading to estimation problems. Consider re-specifying the cutoffs.

Mean Model Estimates (μ)

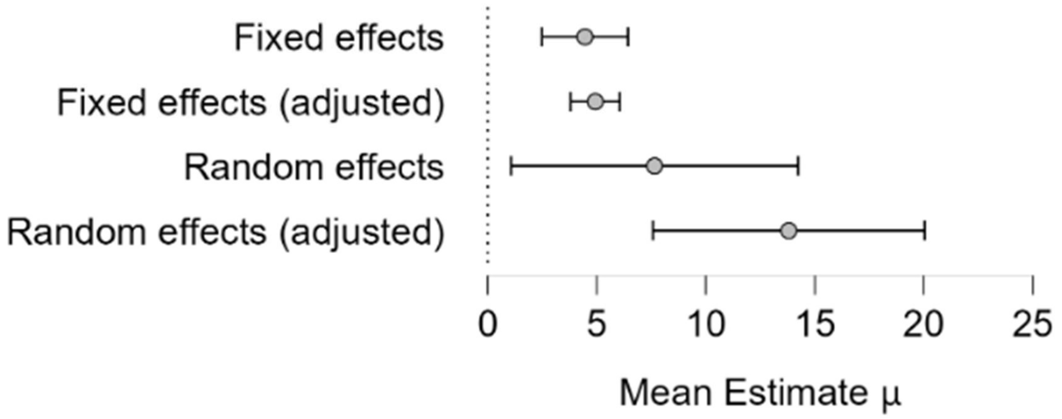

Figure S25. Selection model analysis of sensitivity to publication bias of meta-analysis outcome **PLP expression with native EVs** performed in JASP v 0.98.1 using P-value cut-offs of 0.025 and 0.975 with a fixed effects and random effects inference model: results tables and graph illustrating changes in effect estimate and confidence interval.

Fixed Effects Estimates

Mean Estimates (μ)

	Estimate	Standard Error	z	p	95% Confidence Interval Lower	95% Confidence Interval Upper
Unadjusted	1.073	0.1887	5.685	< .001	0.7029	1.443
Adjusted	1.043	0.01133	92.07	< .001	1.021	1.065

Warning: At least one of the p-value intervals contains no effect sizes, leading to estimation problems. Consider re-specifying the cutoffs.

Random Effects Estimates ▼

Mean Estimates (μ) ▼

	Estimate	Standard Error	z	p	95% Confidence Interval Lower	95% Confidence Interval Upper
Unadjusted	4.373	2.446	1.788	.074	−0.4203	9.167
Adjusted	1.123	0.7595	1.479	.139	−0.3655	2.612

Warning: At least one of the p-value intervals contains no effect sizes, leading to estimation problems. Consider re-specifying the cutoffs.

Mean Model Estimates (μ)

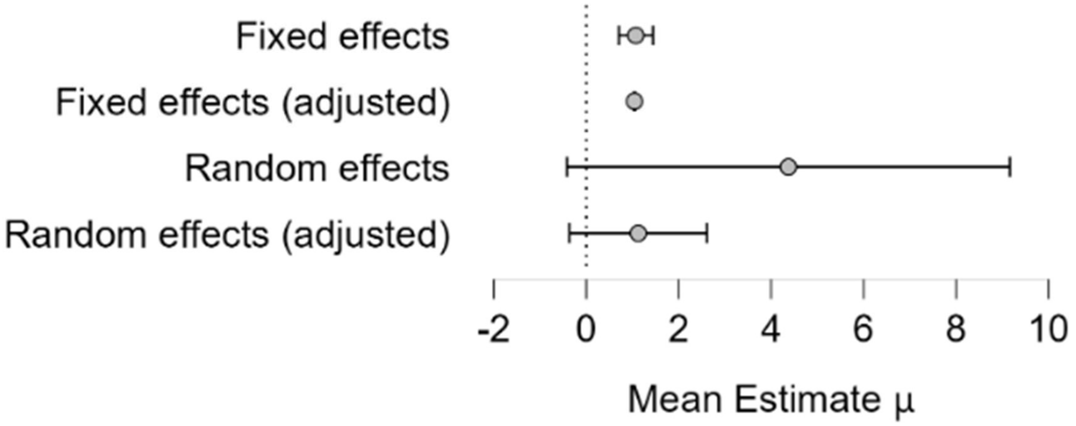

Figure S26. Selection model analysis of sensitivity to publication bias of meta-analysis outcome **Black-Gold myelin % per field with native EVs** performed in JASP v 0.98.1. using P-value cut-offs of 0.025 and 0.975 with a fixed effects and random effects inference model: results tables and graph illustrating changes in effect estimate and confidence interval.
